# KDM3A coordinates actin dynamics with intraflagellar transport to regulate cilia stability

**DOI:** 10.1083/jcb.201607032

**Published:** 2017-04-03

**Authors:** Patricia L. Yeyati, Rachel Schiller, Girish Mali, Ioannis Kasioulis, Akane Kawamura, Ian R. Adams, Christopher Playfoot, Nick Gilbert, Veronica van Heyningen, Jimi Wills, Alex von Kriegsheim, Andrew Finch, Juro Sakai, Christopher J. Schofield, Ian J. Jackson, Pleasantine Mill

**Affiliations:** 1 Medical Research Council Human Genetics Unit, Institute of Genetics and Molecular Medicine, University of Edinburgh, Western General Hospital, Edinburgh EH4 2XU Scotland, UK; 2 Edinburgh Cancer Research UK Centre, Institute of Genetics and Molecular Medicine, University of Edinburgh, Western General Hospital, Edinburgh EH4 2XU Scotland, UK; 3 Department of Chemistry, Chemistry Research Laboratory, OX1 3TA Oxford, England, UK; 4 Division of Metabolic Medicine, Research Center for Advanced Science and Technology, The University of Tokyo, Tokyo 153-8904, Japan

## Abstract

Cilia assembly and disassembly are coupled to actin dynamics, ensuring a coherent cellular response during environmental change. How these processes are integrated remains undefined. The histone lysine demethylase KDM3A plays important roles in organismal homeostasis. Loss-of-function mouse models of *Kdm3a* phenocopy features associated with human ciliopathies, whereas human somatic mutations correlate with poor cancer prognosis. We demonstrate that absence of KDM3A facilitates ciliogenesis, but these resulting cilia have an abnormally wide range of axonemal lengths, delaying disassembly and accumulating intraflagellar transport (IFT) proteins. KDM3A plays a dual role by regulating actin gene expression and binding to the actin cytoskeleton, creating a responsive “actin gate” that involves ARP2/3 activity and IFT. Promoting actin filament formation rescues *KDM3A* mutant ciliary defects. Conversely, the simultaneous depolymerization of actin networks and IFT overexpression mimics the abnormal ciliary traits of *KDM3A* mutants. KDM3A is thus a negative regulator of ciliogenesis required for the controlled recruitment of IFT proteins into cilia through the modulation of actin dynamics.

## Abstract (precis)

Yeyati et al. demonstrate that the histone demethylase KDM3A acts as a negative regulator of ciliogenesis by modulating actin dynamics, both transcriptionally and by directly binding actin. KDM3A influences local actin networks to restrict intraflagellar transport during ciliogenesis; in its absence, cilia become unstable with abnormal lengths and accumulated intraflagellar transport proteins.

## Introduction

Primary cilia are homeostatic sensory organelles driven by intraflagellar transport (IFT) that integrate multiple and diverse cellular inputs critical to normal development and organ homeostasis. Diverse environmental cues affect their assembly and disassembly in a manner that is tightly coupled to the actin cytoskeleton and changes in gene expression. How cells coordinate these processes to effect a coherent cellular response remains unclear.

Actin network architecture is increasingly recognized as a major driver of ciliogenesis in cycling cells (Pitaval et al., 2010). Chemical and genetic perturbations of multiple actin remodeling factors have been found to promote ciliogenesis or affect ciliary length (Kim et al., 2010, 2015; Cao et al., 2012; Hernandez-Hernandez et al., 2013; Kang et al., 2015) and intriguingly rescue ciliogenesis defects in *Ift88* (Kim et al., 2010) and *IFT121* (Fu et al., 2016) mutant cells. Actin-myosin–mediated contraction participates in ciliary abscission as the neuronal differentiation program is triggered (Das and Storey, 2014). Leptin elongates hypothalamic cilia via transcriptional regulation and actin destabilization (Kang et al., 2015), whereas the microRNA *miR-129-3p* regulates cilia assembly through the concomitant transcriptional silencing of centrosomal proteins and repression of actin filament formation (Cao et al., 2012). Altogether, these findings highlight the existence of concurrent regulatory mechanisms coordinating ciliogenesis, actin dynamics, and transcription.

Interestingly, mouse mutants for the histone *N-*lysine demethylase *Kdm3a* (*Jmjd1a*, *Tsga*, or *Jhdm2a*) are obese, predisposed to diabetes (Inagaki et al., 2009; Tateishi et al., 2009), and have male infertility phenotypes (Okada et al., 2007) overlapping with mouse models of dysfunctional cilia. Structural abnormalities of the acrosome and manchette were observed in mutant *Kdm3a* mouse spermatids (Kasioulis et al., 2014) similar to those reported for conditional inactivation of *Ift88* (Kierszenbaum, 2002; San Agustin et al., 2015). KDM3A localizes to the actin-rich acrosome–acroplaxome region, which is altered in *Kdm3a* mutant spermatids (Kasioulis et al., 2014). In spite of these phenotypic and functional overlaps, a link between KDM3A and ciliogenesis has not been investigated.

KDM3A is part of the large family of Jumonji C (JmjC) domain–containing proteins (Clissold and Ponting, 2001; Klose et al., 2006; Markolovic et al., 2016). Deregulated expression of KDM3A is associated with colorectal cancer (Uemura et al., 2010), breast cancer (Wade et al., 2015), and hepatocellular carcinoma (Yamada et al., 2012). Hypoxia (Beyer et al., 2008; Pollard et al., 2008; Wellmann et al., 2008) and the HSP90 chaperone machinery (Kasioulis et al., 2014) are reported to modulate KDM3A levels and activity, whereas its interaction with chromatin is modulated by cold-regulated phosphatases (Abe et al., 2015). Overall, these findings indicate that KDM3A plays fundamental roles in organismal homeostasis, integrating multiple environmental cues with physiological states.

KDM3A presents different regulatory modalities and subcellular distributions. Through the catalytic removal of mono- and *N-*dimethyl groups from lysine 9 of histone H3, KDM3A acts as a transcriptional activator (Yamane et al., 2006) of multiple genes involved in spermatogenesis (Okada et al., 2007), fat storage (Okada et al., 2010), and energy expenditure (Inagaki et al., 2009) as well as of diverse types of cancers (Ohguchi et al., 2016; Zhao et al., 2016). More recently, scaffolding and noncatalytic modes of transcriptional regulation by KDM3A have emerged wherein KDM3A binds to the SWI–SNF (Switch-sucrose nonfermentable) chromatin-remodeling complex (Abe et al., 2015) or Hedgehog-responsive transcription factor GLI1 (Schneider et al., 2015), regulating expression of target genes. Although these functions relate to the role of KDM3A in the nucleus, KDM3A exits the nucleus in response to mechanosensitive stimuli (Kaukonen et al., 2016) and is found at various cytoplasmic sites of somatic cells and during germ cell development (Okada et al., 2007; Yang et al., 2009; Yamada et al., 2012; Kasioulis et al., 2014). Altogether, these findings indicate that KDM3A is a multifunctional protein with highly regulated subcellular distributions and nontranscriptional roles that remain to be explored.

Here, we present evidence on the regulation of actin dynamics by KDM3A and the subsequent impact on IFT and ciliogenesis. KDM3A is required for the stabilization of primary cilia and regulates cilial responsiveness to environmental cues. RNA-sequencing, proteomic, and two-hybrid studies reveal a dual role for KDM3A in this integrated response modulating ciliogenesis. On a global level, it senses extracellular signaling and transcriptionally regulates the free pool of actin available. On a very local level, KDM3A directly binds to the actin cytoskeleton, creating a responsive “actin gate” and regulating IFT within cilia and periciliary compartments. Importantly, deregulated expression of an IFT protein, IFT81, coupled with actin depolymerization phenocopies cilial defects of *KDM3A* mutants, demonstrating that KDM3A-mediated cytoskeletal changes tightly regulate IFT during ciliary growth.

## Results

### KDM3A plays a conserved role in the regulation of ciliary dynamics

**Figure 1.**
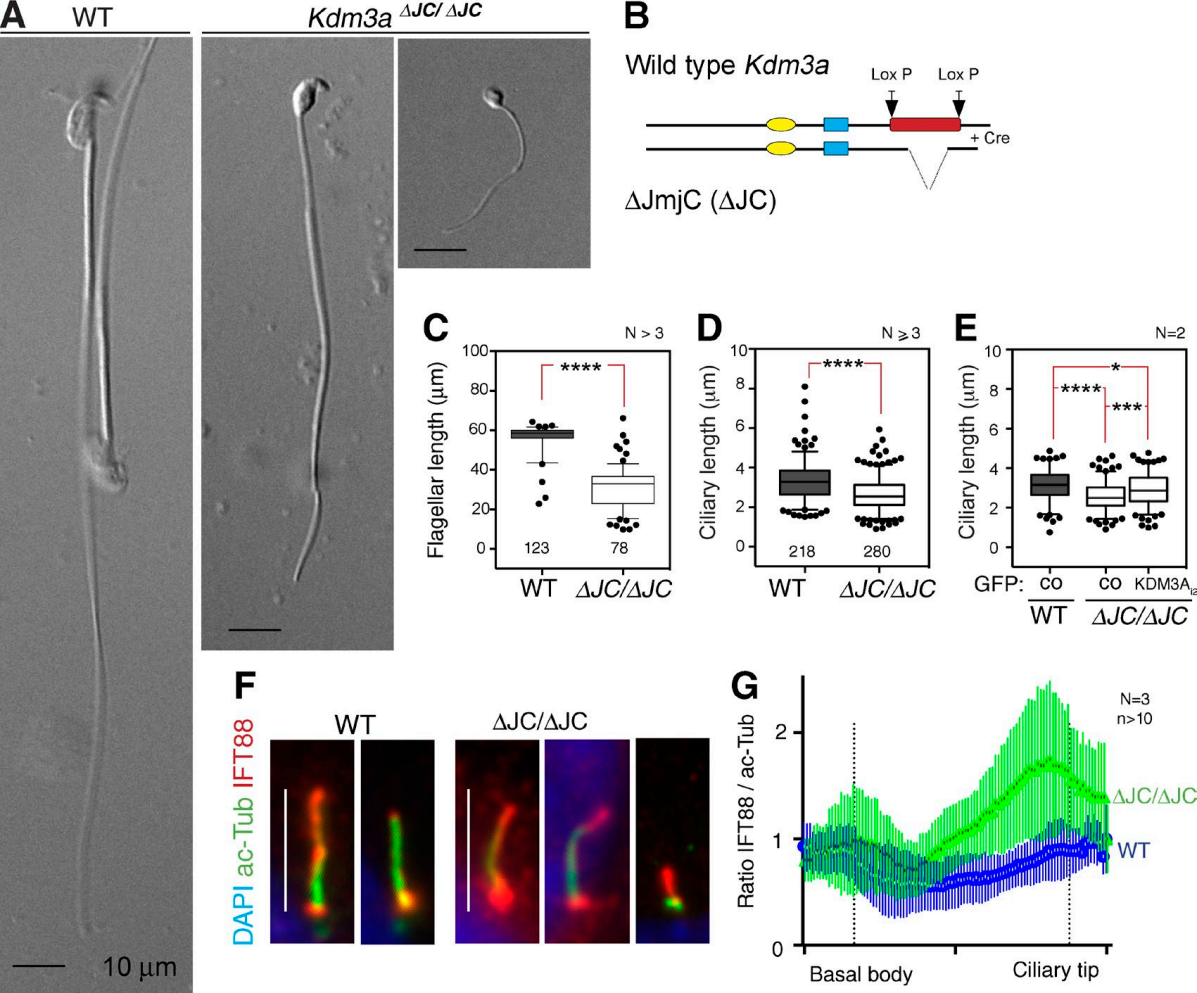
**Axonemal lengths are disrupted in *Kdm3a* mutant mice.** (A) Wild-type (WT) and *Kdm3a^ΔJC/ΔJC^* mutant spermatozoa from mouse epididymis; the latter have rounded heads and short flagella. (B) Schematic of the gene-targeted deletion of *Kdm3a^ΔJC^* mutant mouse model. Domains: yellow, C6-type zinc finger (ZF); blue, LXXLL nuclear receptor binding (NR); red, JmjC domain (JmjC). (C) Mean ± 5th–95th percentile of flagellar lengths of epididymal spermatozoa derived from *N* ≥ 3 per genotype; *n* is indicated below each box. (D) Mean ± 5th–95th percentile of MEF ciliary lengths of MEFs (*N* ≥ 3 per genotype) after 24-h serum depletion. (E) Mean ± 5th–95th percentile of MEF ciliary lengths transfected with GFP vector (control [CO]) or GFP-KDM3A_i2_, showing partial rescue of the mutant short cilia phenotype. (F) Immunostaining illustrates ciliary accumulations of IFT88 in *Kdm3a^ΔJC^* mutant MEFs. (G) Ratio of averaged fluorescence intensity of IFT88 relative to acetylated α-tubulin measured from the basal body to the ciliary tip along ciliary axonemes. Error bars represent ± SEM for >10 cilia per genotype. Asterisk denotes significant p-value from *t* test: *, P < 0.05; ***, P < 0.001; ****, P < 0.0001.

During the analysis of a deletion mutant mouse model of *Kdm3a* lacking the catalytic domain JmjC (ΔJC; Kasioulis et al., 2014), we observed that flagella of mutant spermatids were consistently shorter than those of control littermates (Fig. 1, A–C). We then asked whether KDM3A played a broader role in ciliogenesis, examining primary cilia in somatic *Kdm3a^ΔJC^* cells. Mouse embryonic fibroblasts (MEFs) derived from *Kdm3a^ΔJC^* mutants show abnormal ranges of ciliary lengths (Fig. 1 D), which can be partially rescued by transfecting a short cytoplasmic isoform of KDM3A (Fig. 1 E, KDM3A_i2_-EGFP; Kasioulis et al., 2014). *Kdm3a^ΔJC/ΔJC^* MEFs also displayed accumulations of IFT88 at the ciliary base and tip compared with the uniform distribution of IFT88 in wild-type cilia (Fig. 1, F and G). These observations suggested that KDM3A could play a role in axonemal dynamics and IFT in primary cilia. Because *Kdm3a^ΔJC^* mutant cells encode a truncated KDM3A protein, albeit at very low levels (Kasioulis et al., 2014), we generated *KDM3A^null^* cells in the immortalized human retinal epithelium hTERT RPE1 cell line. RPE1 cells have been thoroughly characterized to study ciliary growth, resorption, and axoneme length in response to cell density and serum content of the culture media (Pugacheva et al., 2007; Kim et al., 2015).

**Figure 2.**
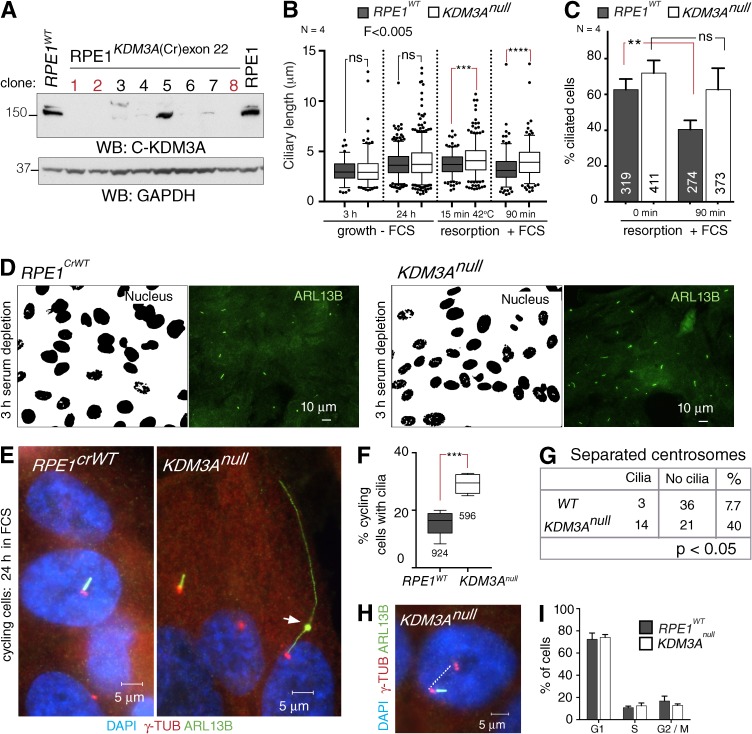
**KDM3A is a negative regulator of ciliogenesis.** (A) Total cell extracts of wild-type (*RPE1^WT^*) and *KDM3A* gene-edited RPE1 mutants (*KDM3A^Cr22^*, named herein *KDM3A^null^*) immunoblotted with KDM3A, revealing *KDM3A*-null clones (1, 2, and 8). (B) Mean ± 5th–95th percentile of ciliary lengths (*N* = 4) of wild-type lines (*RPE1^WT^*, *RPE1^CrWT^*) and the *KDM3A^null^* lines (1,2,8) after 3 h or 24 h without serum (−FCS) and after 24 h −FCS followed by 15-min temperature stress (42°C) in the presence of serum or 90-min serum only. Dots represent cilial lengths outside the indicated percentile (*n* > 100 cilia per experiment). One-way analysis of variance, ****, P < 0.0001; paired *t* test, **, P < 0.01; ***, P < 0.001; ns, not significant. The F-test illustrates the length variance between *RPE1^WTs^* and *KDM3A^nulls^* for each ciliary phase. (C) Percentages of ciliated cells after 24 h −FCS at the indicated times after serum replenishment. (D) Segmented nuclei and cilia (stained with anti-ARL13B) of *RPE1^CrWT^* and *KDM3A^null^* (line 1) cultures after 3-h serum depletion highlights differences in the frequency of ciliated cells (41%, SD = 7.6; 64%, SD = 7.02, respectively), in spite of identical cell densities. Percentages are from *n* > 100 cells per experiment done in triplicate. χ^2^ < 0.001. (E) Wide-field images illustrating the extreme range of lengths often observed in exponentially growing *KDM3A^null^* lines (24 h FCS), with frequent abnormal ciliary bulges in mutants (arrow). (F) Percentage of cycling cells (24 h +FCS) that contain a cilium in control (*RPE1^WT^* and *RPE1^CrWT^*) and *KDM3A^null^* (lines 2 and 8) cells (*N* = 3). ***, P = 0.001 is from χ^2^ test. (G) Scoring of cilia found in late G2 (24 h +FCS or during serum induced ciliary resorption). WTs represent *RPE1^WT^* and *RPE1^CrWT^*, and *KDM3A^null^* lines 1, 2, and 8. P-value is from χ^2^ test. (H) Example of a mutant cilium remaining after centrosomes have separated (>2 µm). (I) Cell cycle profile of cycling (24 h +FCS) control (*RPE1^WT^* and *RPE1^CrWT^*) and *KDM3A^null^* (lines 1, 2, and 8) cultures. Error bars represent SD.

**Figure 3.**
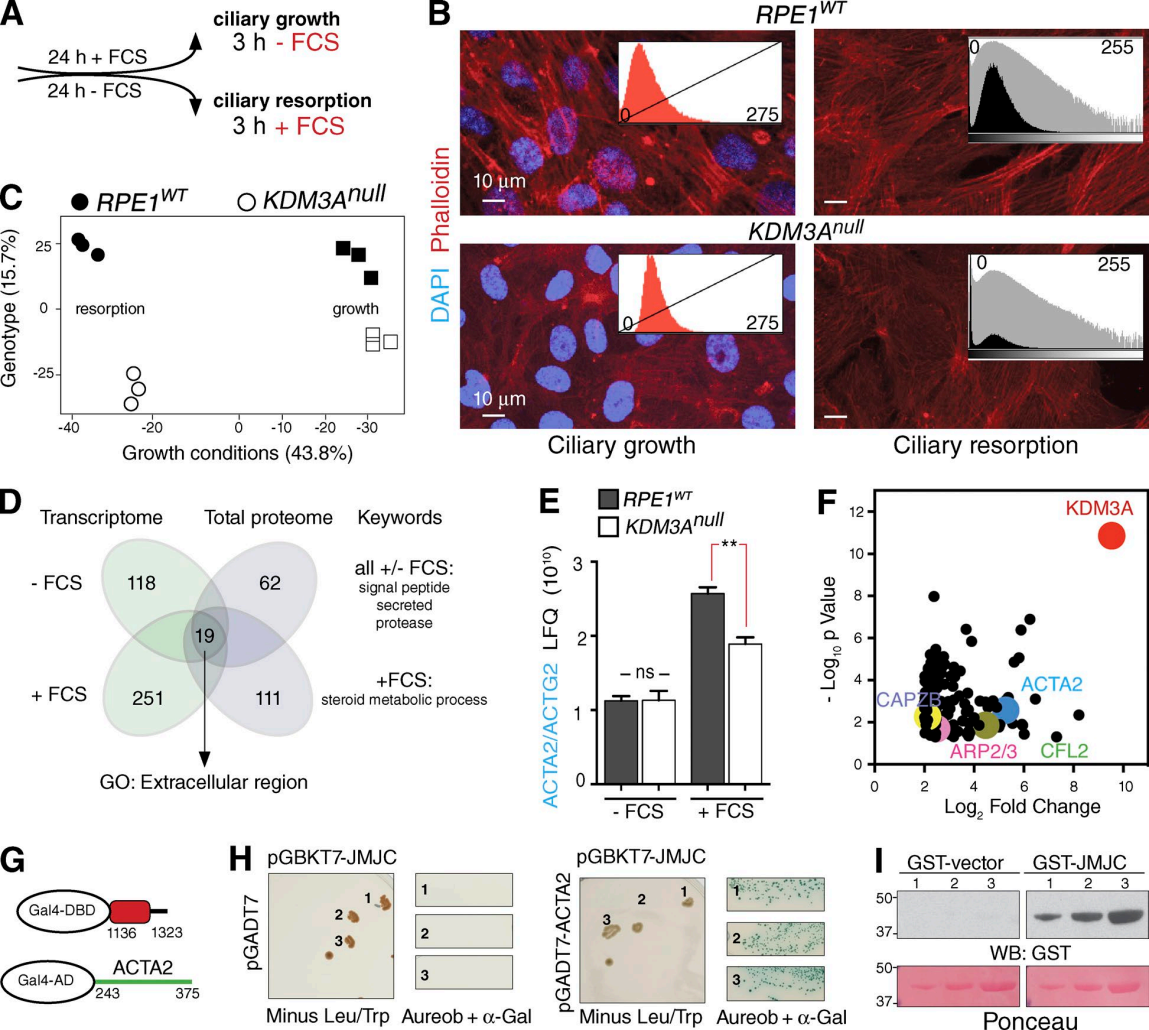
**KDM3A regulates the expression of and interacts with actin components.** (A) Schematic of experiments used to isolate RNA and protein lysates during ciliary growth and resorption. (B) Phalloidin staining and histograms of pixel intensities reveal decreased abundance of stress fibers in *KDM3A^null^* cells. (C) Principal-component analysis of total cell proteomes from *RPE1^WT^* and *KDM3A^null^* cells during ciliary growth and resorption show that variability between growth conditions (43.8%) is larger than that between genotypes (15.7%). (D) Venn diagram illustrates the number of significantly down-regulated genes in *KDM3A^null^* compared with *RPE1^WT^* in the transcriptomic and proteomic datasets during ciliary growth and resorption. Dysregulation of extracellular environment is the most enriched term by GO analysis (Table S4). (E) Label-free quantification (LFQ) intensities for peptides specific to ACTA2 and ACTG2 (nonseperable because of sequence homology) across the indicated growth conditions obtained from total cell proteomes of control (*RPE1^WT^* and *RPE1^CrWT^*) and *KDM3A^null^* (line 2) lines. **, P < 0.001; ns, not significant. (F) Proteomic-based identification of endogenous components significantly enriched in KDM3A pull-downs during ciliary growth (3 h −FCS). P-values are from *t* tests of label-free quantifications from *RPE1^WT^* relative to *KDM3A^null^* from three experimental replicates. Select isolated actin-binding proteins are highlighted. (G) Schematic of the KDM3A region used as bait (JMJC domain in red) and ACTA2 prey identified in the two-hybrid screen. (H) Independent colonies (one to three) of rescued bait/prey plasmid cotransformed onto two-hybrid Gold yeast and grown in double selection (Leu/Trp) were resuspended and assessed for aureobasidin A resistance and α-Gal activity. Only yeast cotransformed with JmjC and ACTA2 grew in the stringent conditions indicating a binary interaction. (I) Pure smooth muscle actin resolved in 4–12% acrylamide gel (three serial dilutions) transferred to nitrocellulose is bound by GST-JMJC, but not GST.

Using CRISPR/Cas9 gene editing directed to exon 22 of *KDM3A*, we generated independent RPE1 cell lines null for *KDM3A*. Lines 1, 2, and 8 (*KDM3A^null^*) did not express KDM3A protein (Fig. 2 A) and were found to bear compound *KDM3A* mutations by genotyping (Fig. S1, A and B). As controls, we used the parental RPE1 line (*RPE1^WT^*) as well as an RPE1 sister line that has undergone CRISPR/Cas9 editing but was wild type for *KDM3A* (*RPE1^CrWT^*). Cells were plated at equal densities and promoted either to (a) ciliate, by serum withdrawal (−FCS), or (b) resorb cilia, by serum replenishment (+FCS), with or without temperature stress. No differences were observed in the cilial length between controls and *KDM3A* null cultures after 3 or 24 h without serum (Fig. 2 B, 3 h and 24 h −FCS). In contrast, under resorption conditions, *KDM3A^null^* cilia remained significantly longer than those of controls (Fig. 2 B, resorption +FCS), suggesting that *KDM3A^null^* cells failed to efficiently resorb cilia. Moreover, the proportion of *KDM3A^null^* cells that remained ciliated was unchanged after serum replenishment, in contrast to the efficient resorbtion of ciliated control cells (Fig. 2 C).

It was noted during these studies that *KDM3A^null^* cilia displayed a wider range of lengths than *RPE1^WT^* under all conditions (Fig. 2 B, analysis of variance F). Also 3 h after serum withdrawal, *KDM3A^null^* cultures had a higher proportion of ciliated cells than controls (Fig. 2 D). Together, these results suggest that mammalian KDM3A negatively regulates ciliogenesis.

Ciliogenesis is usually promoted through serum withdrawal, but cycling RPE1 cells also form cilia in the presence of serum, albeit at lower frequencies (Cao et al., 2012). To confirm the propensity of *KDM3A^null^* cells to ciliate, we examined ciliogenesis in exponentially growing cultures. In the presence of serum, some extremely long cilia with abnormal bulges were observed in *KDM3A^null^* cells (Fig. 2 E, arrow). Importantly, higher proportions of ciliated cells were consistently observed in mutants (Fig. 2 F), in spite of comparable cell density and mitotic index to controls (*RPE1^WT+CrWT^* 4.05%, SD = 1.90; *KDM3A^null^* 6%, SD = 1.63; *N* = 3 biological replicates, *n* > 300 cells scored/condition). A higher proportion of *KDM3A^null^* cells retained cilia into late G2 (Fig. 2, G and H), whereas most wild-type cilia were already resorbed (Spalluto et al., 2013). However, these changes were neither caused by nor associated with alterations in cell cycle progression (Fig. 2 I). As we did not observe mutant mitotic cells with cilia, we conclude that mutant cilia must be eventually resorbed albeit with altered kinetics.

Both *Kdm3a^ΔJC^* deletion and *KDM3A^null^* mutant models indicate that KDM3A plays a role in axoneme dynamics, yet the deletion mutants have on average shorter cilia (Fig. 1 D) rather than the wider range of lengths observed in *KDM3A^null^* cells (Fig. 2 B). These contrasting outcomes may be caused by a gain of function of the truncated KDM3A protein encoded by *Kdm3a^ΔJC^* mutants, as MEFs derived from a second hypomorphic mouse model of *Kdm3a* (*Kdm3a^GT^*; Kasioulis et al., 2014) present, as observed in *KDM3A^null^* RPE1 cells, a wider range of cilial lengths (Fig. S1, C and D). Ciliary resorption in response to serum replenishment and temperature stress was similarly delayed in *Kdm3a^GT/GT^* MEFs (Fig. S1 E).

In summary, given the increased proportion of ciliated cells, abnormal range of cilial lengths, and delayed cilial resorption kinetics, our loss-of-function mutants suggest that in the absence of KDM3A, ciliogenesis is facilitated and may be less tightly regulated. Conversely, overexpressing KDM3A reduces cilia formation in the parental *RPE1^WT^* line (Fig. S1, F and G).

### KDM3A regulates actin expression and binds actin in vivo and in vitro

Given KDM3A’s localization to actin-rich structures and altered biochemical properties of actin in *Kdm3a* mutant spermatids (Kasioulis et al., 2014), we hypothesized that the effect of KDM3A depletion on ciliogenesis is a consequence of actin dysregulation. As KDM3A had been shown to bind the promoter and regulate the expression of smooth muscle α-actin (*Acta2*; Lockman et al., 2007), these effects could be caused by altered transcription or protein–protein interactions or both. Indeed, phalloidin staining of *KDM3A^null^* cells (Fig. 3 A) showed consistently decreased abundance of actin filaments compared with *RPE1^WT^* (Fig. 3 B), and this was also evident in *Kdm3a^ΔJC/ΔJC^* MEFs (Fig. S2 A).

The turnover of the actin cytoskeleton is both transcriptionally and posttranscriptionally regulated (Olson and Nordheim, 2010), requiring a pool of actin monomers (G-actin) and multiple actin-remodeling factors that determine F-actin formation (Blanchoin et al., 2014). We therefore investigated both transcriptional changes and protein–protein interactions that could contribute to KDM3A ciliary traits. To address the global transcriptional changes that occur in the absence of KDM3A during ciliary growth and resorption, we used a combined approach of RNA sequencing and quantitative label-free mass spectrometric analysis (Tables S2 [raw datasets] and S3 [thresholds]). Principal-component analysis of *RPE1^WT^* and *KDM3A^null^* proteomes first showed that differences between samples primarily reflected serum content of the culture media as opposed to genotypes of the cell lines (Fig. 3 C). This supports previous studies reporting that KDM3A does not cause large global transcriptional changes but is instead a fine regulator of gene expression (Okada et al., 2007). Subsequently, considering that KDM3A is a transcriptional activator (Yamane et al., 2006), we identified genes for which protein or RNA abundance were reduced in *KDM3A^null^* cells compared with *RPE1^WT^* controls (Table S3). GO(Gene Ontology) analysis suggests that the transcriptional activity of KDM3A is required to sense the extracellular environment (Fig. 3 D and Table S4). In agreement with previous findings (Lockman et al., 2007), direct interrogation of actin expression in these datasets reveals that smooth α-actin *ACTA2* expression (Table S3) as well as protein levels (Fig. 3 E) failed to be up-regulated in response to serum replenishment. A reduced abundance of ACTA2 in mutants could potentially compromise actin polymerization by limiting available actin monomers during ciliary resorption (+FCS). However, as actin levels are normal in *KDM3A^null^* cells in the absence of serum, transcriptional control alone cannot explain perturbations of actin dynamics observed in mutant cells during ciliary growth (−FCS).

Next, we investigated a potentially direct effect of KDM3A on the cytoskeleton through protein interactions using KDM3A immunopurification of total cell extracts followed by mass spectrometry. In parallel, we performed a subsaturation two-hybrid screen using the JMJC domain of KDM3A as bait. The KDM3A antibody significantly enriched 166 proteins from *RPE1^WT^* (Fig. 3 F and Table S3), where GO analysis revealed actin binding and actin cytoskeleton as the most enriched terms (Table S4). Strikingly, these immunopurifications showed that endogenous KDM3A copurified with ACTA2 and the actin-capping protein CAPZB, both of which were also found in the two-hybrid screen. A binary interaction between ACTA2 and the JMJC domain of KDM3A was observed by yeast two-hybrid screening (Fig. 3 H), but not with CAPZB (Fig. S2 B). Further confirmation of a direct association between KDM3A and ACTA2 was supported through binding of purified GST-JMJC to pure smooth muscle α-actin (Figs. 3 I and S3 C). The multiple actin-related proteins that copurified with endogenous KDM3A may thus be indirectly enriched through KDM3A binding to actin filaments.

In summary, global analysis of the transcriptional and total proteomic profiles of cells lacking KDM3A show enrichment of GO terms that relate to the extracellular environment that are in line with the increasingly recognized role of KDM3A in environmental adaptation and mechanosensation (Chakraborty et al., 2016; Kaukonen et al., 2016; Pedanou et al., 2016). Immunopurification of endogenous KDM3A, two-hybrid screening, and far–Western blotting revealed that KDM3A interacts with actin in vivo and in vitro. Together with the transcriptional failure of *KDM3A^null^* cells to induce actin expression in response to serum replenishment, these results reveal a novel role for KDM3A in the modulation of actin dynamics that may underpin phenotypic changes of *KDM3A^null^* cells.

### KDM3A modulates actin dynamics

To characterize the functional interaction between KDM3A and actin, we further investigated cellular phenotypes known to depend on actin dynamics.

**Figure 4.**
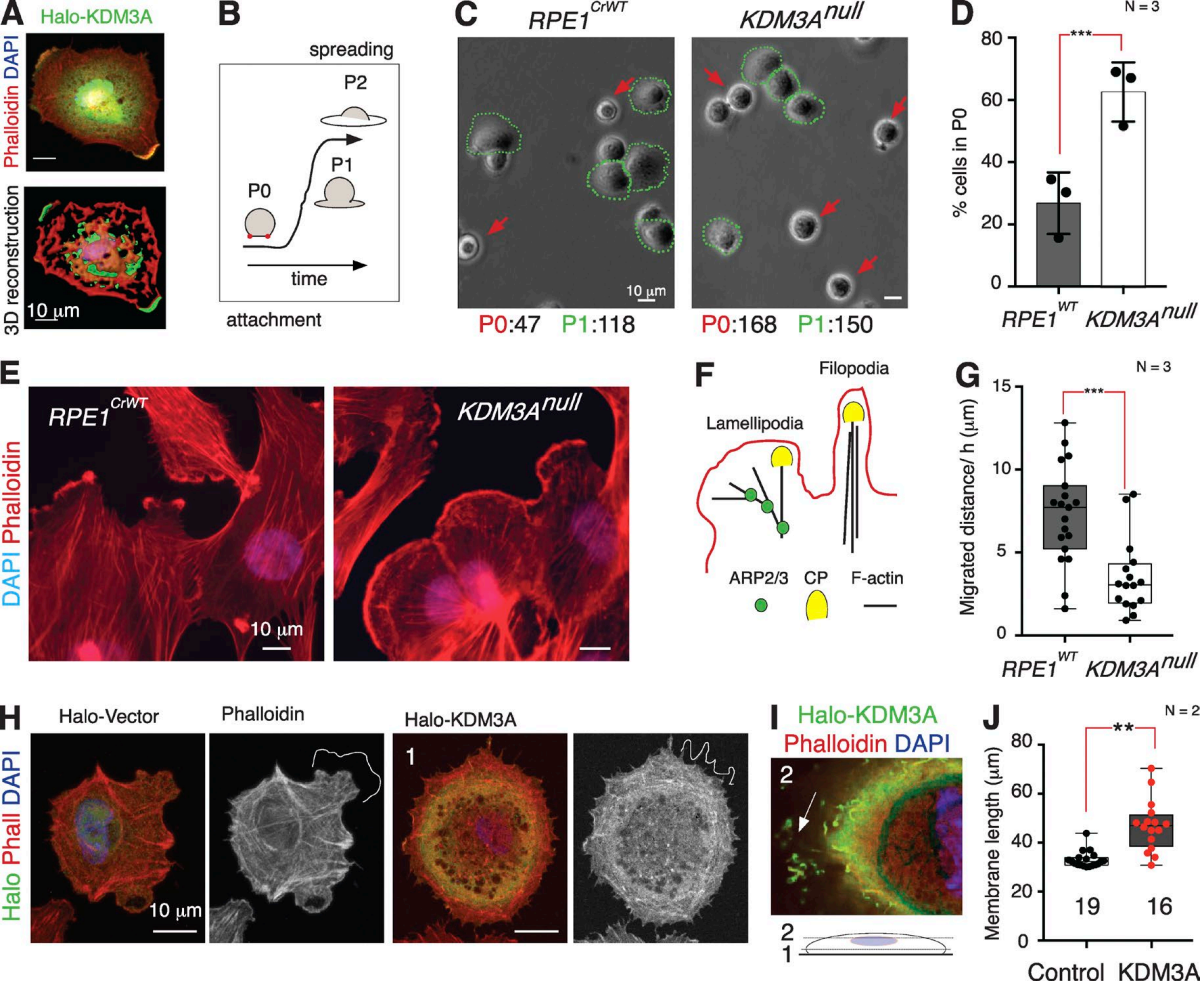
**KDM3A is required for actin polymerization.** (A) 60 min after plating on glass coverslips, 3D reconstruction reveals cytoplasmic distribution of Halo-KDM3A with strong presence at cellular edges and apical cap. (B) Schematic of stages requiring fast actin polymerization occurring from initial attachment (Phase 0 [P0]) to early spreading (P1). P2 represents fully spread cells. (C) Bright-field image and number of *RPE1^WT^* and *KDM3A^null^* cells that remain round (P0) or began spreading (P1) over plastic plates after suspension in the absence of serum for 2 h. Red arrow and green encircling indicate cells at P0, defined by birefringence at edges, and P1, defined by flattened edges, respectively. (D) Proportion of cells that remain in P0 when cultures are plated immediately after trypsinization (*N* = 3, *n* > 50 cells scored/genotype). Error bars: mean ± SD. *, P < 0.0001, from χ^2^. (E) Wide-field images illustrate differences in cellular edges between *RPE1^crWT^* and *KDM3A^null^*. (F) Schematic of actin filament capping or branching leading to changes in cell morphology. As KDM3A binds actin, the rounder cellular edges of *KDM3A* cells suggest an imbalance between capping (yellow) and ARP2/3 mediated branching (green). (G) Median and range of the distance migrated by attached *RPE1^WT^* cells and *KDM3A^null^* (lines 1, 2, and 8; 4 h after plating). Dots represent measurement of distance moved by a single cell. . (H) Confocal images illustrate the distinct cellular edges of *RPE1^WT^* cells transfected with Halo vector or Halo-KDM3A. (I) Hyperfilopodia-like projections are most prominent at the apical pole of Halo-KDM3A transfected cells (plane 2 in diagram). (J) Median and range of membrane lengths at cellular edge of Halo transfected cells measured as described in Fig. S2 D. *N* = 2 transfections. Statistics from G and J are from unpaired *t* tests.

It was recently shown that KDM3A expression is up-regulated when cells are in suspension (Pedanou et al., 2016) and that its cytoplasmic localization depends on mechanosensitive signals (Kaukonen et al., 2016). Indeed, Halo-tagged KDM3A is cytoplasmic and enriched in cortical actin filaments and cellular edges immediately after attachment of suspended cells (Fig. 4 A). We reasoned that the fast wave of actin polymerization (Fig. 4 B) that follows early spreading over solid substrates (Wolfenson et al., 2014) would thus provide functional evidence on the impact of cytoplasmic KDM3A on actin dynamics. Wild-type (*RPE1^WT^* and *RPE1^CrWT^*) and *KDM3A^null^* (lines 1, 2, and 8) cultures were trypsinized and maintained in suspension in serum-free media for 2 h before plating (Fig. 4 C) or plated directly (Fig. 4 D) onto plastic plates. We found that in both cases, a greater proportion of *KDM3A^null^* cells remained unspread compared with control cultures (Fig. 4, C and D; P0/P1 ratio χ^2^ = 0.001), indicating that loss of KDM3A interferes with the early stages of cell spreading that require fast actin polymerization.

In the presence of serum, *KDM3A^null^* cells consistently have rounder cellular edges compared with *RPE1^WT^* (Fig. 4 D). Branching or elongation of actin fibers is dynamically driven by the antagonism between ARP2/3 nucleating activity and capping proteins, ultimately determining the length of the actin fiber (Edwards et al., 2014). At the cellular edge, this interplay is manifested as the extent of lamellipodia to filopodia formation (Fig. 4 E), where the underlying actin-remodeling events steer cell migration (Pollard and Borisy, 2003; Koestler et al., 2013). Consistent with these findings, *KDM3A^null^* cells presented reduced cell motility (Fig. 4 F) and prominent leading edges (Fig. S2 D). Conversely, overexpressing KDM3A increases the proportion of cellular membrane ending in spike/filopodia-like protrusions (Fig. 4, H–J; and Fig. S2 E).

Together, the morphological and behavioral features found in the absence or overexpression of KDM3A demonstrate that KDM3A modulates global actin dynamics and suggest that KDM3A binding to actin may compete with or facilitate the binding of different actin-remodeling proteins.

### Altered actin dynamics underlies the ciliary traits of *KDM3A*-null cells

To confirm that a deregulated actin cytoskeleton in the absence of KDM3A underlies the abnormal ciliary phenotypes observed in mutants, we aimed to rescue these phenotypes using chemical modulators of F-actin formation. Depolymerization of F-actin promotes ciliary growth (Kim et al., 2010, 2015). Conversely, actin polymerization is thought to be required for ciliary resorption (Liang et al., 2016).

**Figure 5.**
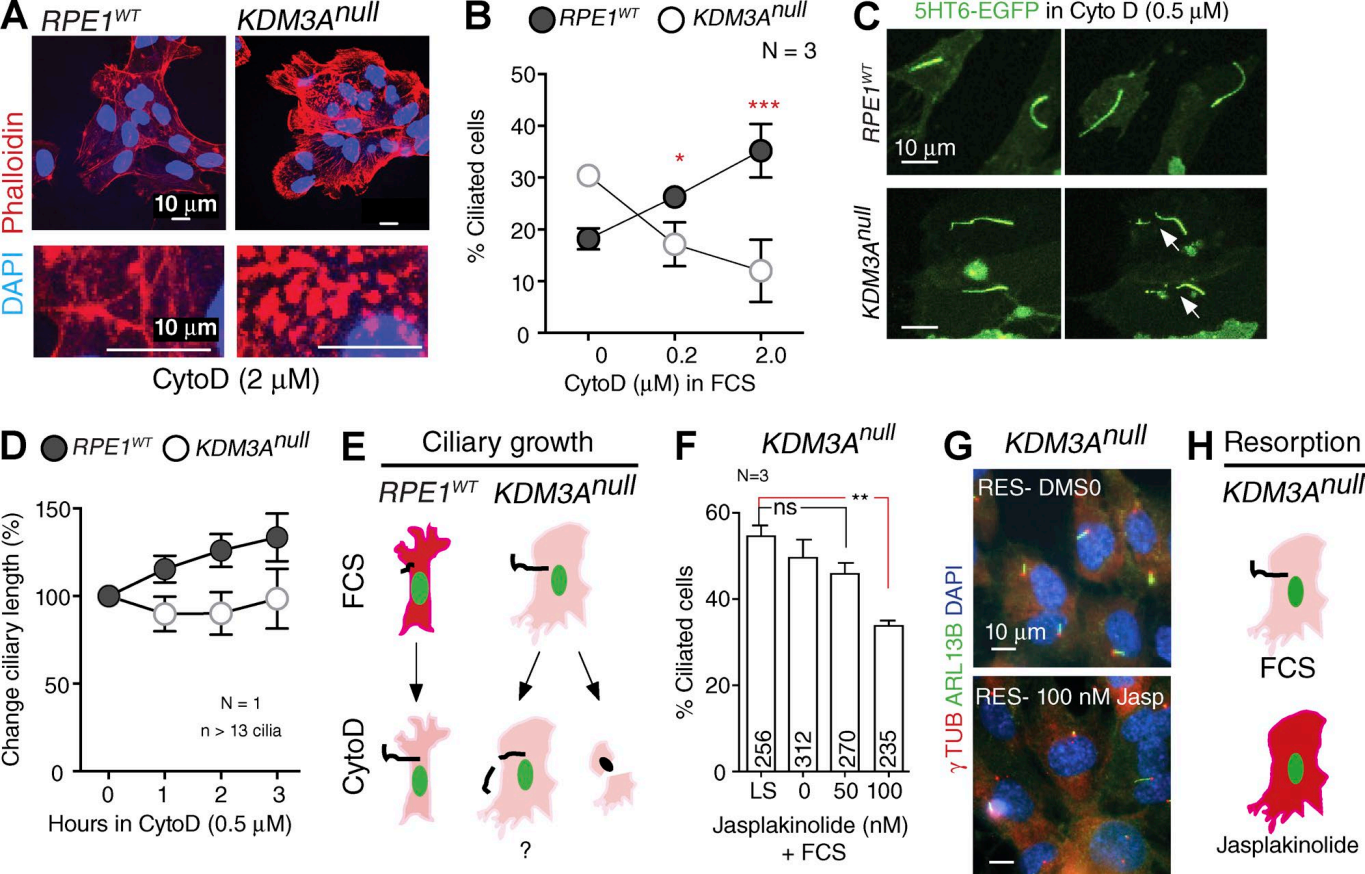
**Altered actin polymerization underlies *KDM3A* mutant ciliary traits.** (A) Wide-field images showing phalloidin-stained foci in *KDM3A^null^* (line 2) when treated with 2 µM CytoD for 4 h +FCS. (B) Percentage of ciliated cycling cells in *RPE1^WT^* and *KDM3A^null^* (line 2) cultures in the presence of CytoD. Symbols represent the mean ± SD. Lines between symbols were added to ease visualization but are not in scale. (C) Single time frames (spanning ∼2 h) from live videos of *RPE1^WT^* and *KDM3A^null^* expressing 5HT6-EGFP in the presence of CytoD and FCS show instable mutant cilia (arrows). (D) Relative increase in ciliary lengths observed during 4 h of live imaging of *RPE1^WT^* and *KDM3A^null^* in the presence of CytoD and FCS. (E) Graphical summary: Depolymerizing actin increase ciliogenesis in cycling *RPE1^WT^* cells. Conversely due to the already reduced number of actin fibers in *KDM3A^nulls^* cells further depolymerization may induce ciliary instability and synthetic lethality. (F) Percentage of ciliated cells after resorption is induced by serum replenishment in the absence (DMSO) or presence of jasplakinolide for 80 min. Error bars represent SD (*N* = 3, number of cells scored indicated within each bar). *, P < 0.05; **, P < 0.01; ***, P < 0.001; ns, not significant. (G) Images of cultures scored in F. (H) Graphical summary of jasplakinolide results.

Interfering with actin dynamics through cytochalasin D (CytoD) treatment promotes ciliogenesis in cycling RPE1 cells without the requirement of serum withdrawal (Cao et al., 2012). Indeed, CytoD treatment alone is sufficient to rescue ciliogenesis in genetic mutants affecting IFT machinery (Kim et al., 2010; Fu et al., 2016). Surprisingly, we instead observed that *KDM3A^null^* cells accumulated abnormal phalloidin-stained foci (Fig. 5 A, 2 µM CytoD) and have decreased proportion of ciliated cells (Fig. 5 B). Live-cell imaging with a ciliary marker (5-HT6-GFP; Berbari et al., 2008) suggests that the decreased proportion of *KDM3A^null^* ciliated cells in 0.5 µM CytoD is likely caused by reduced ciliary stability, as cilia break off the cell body (Fig. 5 C), without the net increase in ciliary length observed in control cultures (Fig. 5 D). CytoD treatment of cycling *RPE1^WT^* cultures thus phenocopies the increased proportion of ciliated cells found in *KDM3A^null^* cells in the presence of serum (Fig. 2 E). The low abundance of actin fibers of *KDM3A* mutants likely facilitates ciliogenesis, but further perturbations in actin polymerization are not tolerated by *KDM3A^null^* cells (Fig. 5 E).

Critically, the converse experiment, where we promoted actin polymerization using jasplakinolide treatment (Kustermans et al., 2008) in the presence of serum, rescued the abnormal resorption of *KDM3A^null^* cilia (Fig. 5, F and G). This suggests that failure of *KDM3A^null^* cells to polymerize actin in response to serum contributes to the abnormal resorption kinetics of mutant cilia (Fig. 5 H).

These pharmacogenetic results support a functionally relevant association between KDM3A and the actin cytoskeleton during ciliogenesis. In the absence of KDM3A, the formation of actin filaments is perturbed and underlies the ciliary defects of *KDM3A^null^* cells.

### A requirement for KDM3A in cilial stability and IFT

**Figure 6.**
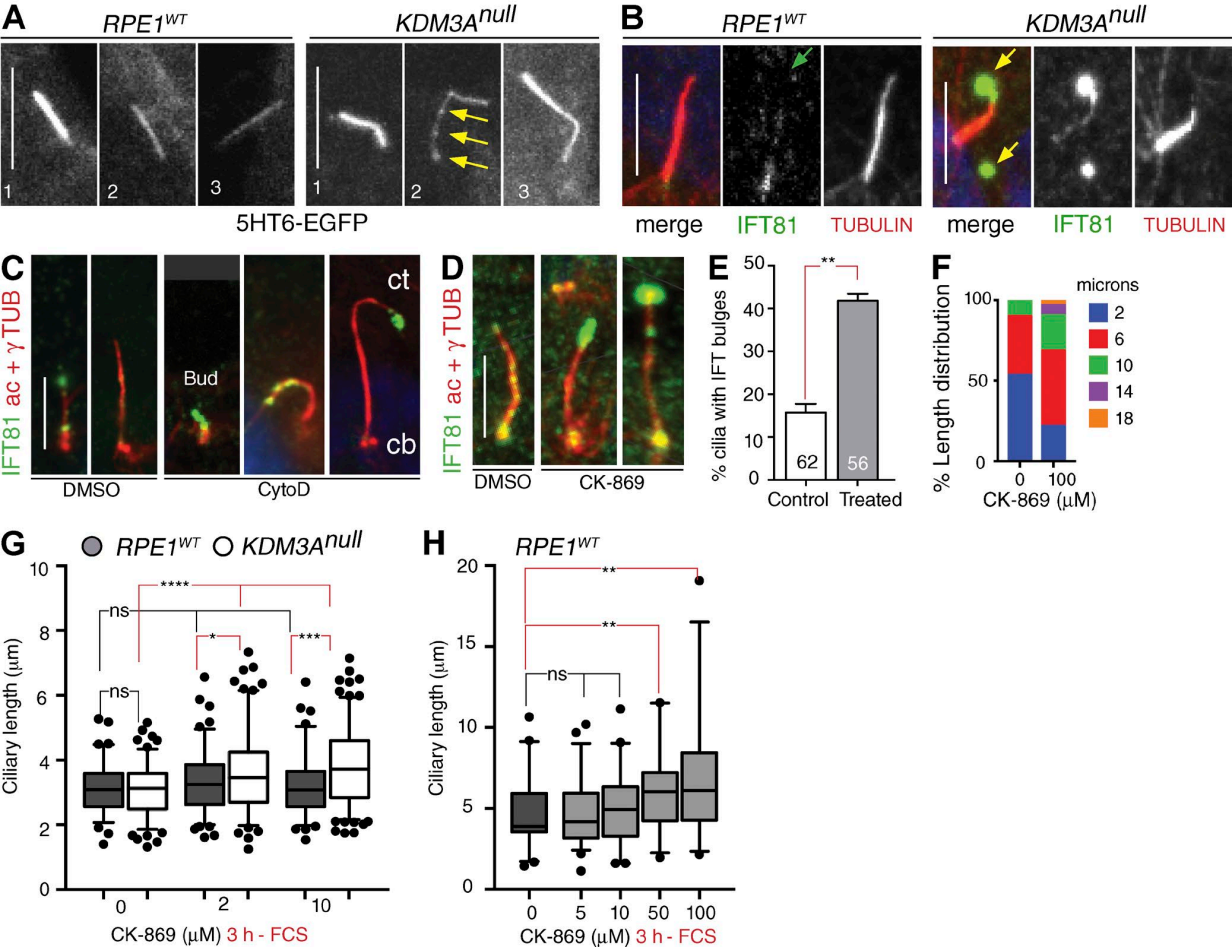
**Perturbing actin polymerization phenocopies IFT abnormalities of *KDM3A* mutants.** (A) Single time frames from Videos 1 and 2 of live *RPE1^WT^* and *KDM3A^null^* (line 2) cultures. Arrows point to bulges forming along *KDM3A^null^* cilia. (B) Confocal images after 48 h −FCS indicate low levels of IFT81 at tip of *RPE1^WT^* cilia (green arrow) but conspicuous accumulations in *KDM3A^null^* mutant cilia, frequently accompanied by IFT81 containing “vesicles” (yellow arrows). (C) Wide-field images of *RPE1^WT^* cultures in the presence of serum show accumulations of endogenous IFT81 in CytoD treated cells that appear sequentially as ciliary buds (cb; without acetylated α-tubulin–stained axonemes, most frequent with 0.2 µM CytoD) and progress toward ciliary tip (ct; 2 µM CytoD). (D) Representative images of IFT81 distribution in cilia formed after 3 h with solvent control or 100 µM CK-869 −FCS. (E) Quantification of cilia with or without IFT81 bulges among treated (2 µM CytoD and 100 µM CK-869) and solvent controls. Numbers in bars indicate number of cilia scored. Error bar represents SD. P-value is from χ^2^ test. (F) Frequency of length ranges illustrate increased proportion of long cilia in 100 µM CK-869–treated cultures. (G) Differential sensitivity of *RPE1^WT^* and *KDM3A^null^* to ARP2/3 inhibition as illustrated by the length of cilia in subconfluent cultures (50–70 cells/0.1 mm^2^) treated for 3 h with 2 and 10 µM CK-869 −FCS. Mean ± 5th–95th percentile (*N* = 3 replicates, *n* > 30 cilia measured/condition). (H) Mean ± 5th–95th percentile of ciliary lengths indicate that *RPE1^WT^* cultures begin to show significant cilial elongation in ≥50 µM CK-869 (*N* = 3 replicates, *n* > 30 cilia measured/condition). *, P < 0.05; **, P < 0.01; ***, P < 0.001; ****, P < 0.0001 (paired *t* test); ns, not significant. Bars, 5 µm.

Live imaging suggested that *KDM3A^null^* cilia were unstable. We investigated whether this instability was caused by the drug treatment or whether it is an inherent property of mutant cilia. Live imaging of cell during ciliary growth (−FCS) revealed that *RPE1^WT^* cilia remained fairly stable, whereas *KDM3A^null^* cilia underwent excessive elongation and fragmentation (Videos 1 and 2 and Fig. 6 A). Similar ciliary instability was observed in *Kdm3a^ΔJC^* mutant MEFs (Video 3). These results suggest that instability of mutant axonemes in the absence of KDM3A reflect an inability to regulate axonemal length. These findings are supported by the unresponsiveness of *KDM3A^null^* cilia to resorb (Video 4) and the wider range of lengths observed in fixed *KDM3A* mutant cilia (Fig. 2).

The presence of ciliary bulges (Fig. 2 E) and their shedding observed during live imaging of *KDM3A^null^* cells suggest that the instability of *KDM3A* mutant cilia results from perturbation of IFT. Indeed, IFT staining of MEFs (Fig. 1 F) and RPE1 null for KDM3A (Fig. 6 B) showed abnormal distributions of IFT88 and IFT81. The anomalies were most evident at the ciliary tip (Fig. 6 C) but could also be observed at the ciliary base (Fig. 1 F).

In *Chlamydomonas reinhardtii,* actin is required for the recruitment of IFT particles to the basal body, where it controls flagellar entry of IFT trains (Avasthi et al., 2014). Accordingly, the abnormal distribution of IFTs observed in *KDM3A^null^* cells could be caused by the permissive and depolymerized state of the actin cytoskeleton in these mutants. If this was true, then perturbing actin dynamics in *RPE1^WT^* would phenocopy the IFT anomalies observed in *KDM3A* mutants. To test this, we treated cells with CytoD or CK-869, a small molecule that specifically inhibits ARP2/3 complex components (Hetrick et al., 2013) copurifying with KDM3A (Fig. 3 F). Perturbations of ARP2/3 activity could contribute to themorphological phenotype of *KDM3A^null^* cells (Fig. 4, E–G). *RPE1^WT^* cells treated with either drug in the presence or absence of serum showed large accumulations of IFT81 at the ciliary tip of *RPE1^WT^* cells (Fig. 6, C–E) accompanied by ciliary elongation (Fig. 6 F). The effect of CK-869 was particularly interesting, as *KDM3A^null^* cells were extremely responsive to ARP2/3 inhibition, showing ciliary elongation from doses as low as 2 µM (Fig. 6 G), whereas *RPE1^WT^* cells only respond from 50 µM (Fig. 6 H). This functional synergism between *KDM3A* mutants and CK-869 treatment indicates a common underlying mechanism by which KDM3A influences IFT through actin polymerization involving the ARP2/3–actin assembly pathway.

In summary, live imaging studies provide evidence that in the absence of KDM3A, cilia become unstable. Antibody staining showed abnormal distributions of endogenous IFTs in mutant cilia, and this trait can be phenocopied in wild-type cells by perturbing actin dynamics. Using two different drugs interfering with actin nucleation, we demonstrate that in mammalian primary cilia, actin dynamics also modulates IFT. Altogether, these findings provide evidence that in the absence of KDM3A, actin dynamics are perturbed, disrupting IFT and destabilizing cilia.

### *KDM3A^null^* cilia reveal an actin-driven constraint for IFT in wild-type cilia

The finding that inhibiting actin polymerization in mammalian control cells phenocopies the abnormal IFT loaded bulges found in *KDM3A^null^* cells suggests that wild-type cilia have an actin-dependent constraint that modulates ciliary IFT. To test this further, we increased IFT protein levels by transfecting tagged IFT81 into *RPE1^WT^* and *KDM3A^null^* cells, reasoning that if an actin gate does exist and is dysfunctional in the absence of KDM3A, IFT overexpression would have opposing effects in wild-type and *KDM3A* mutants.

**Figure 7.**
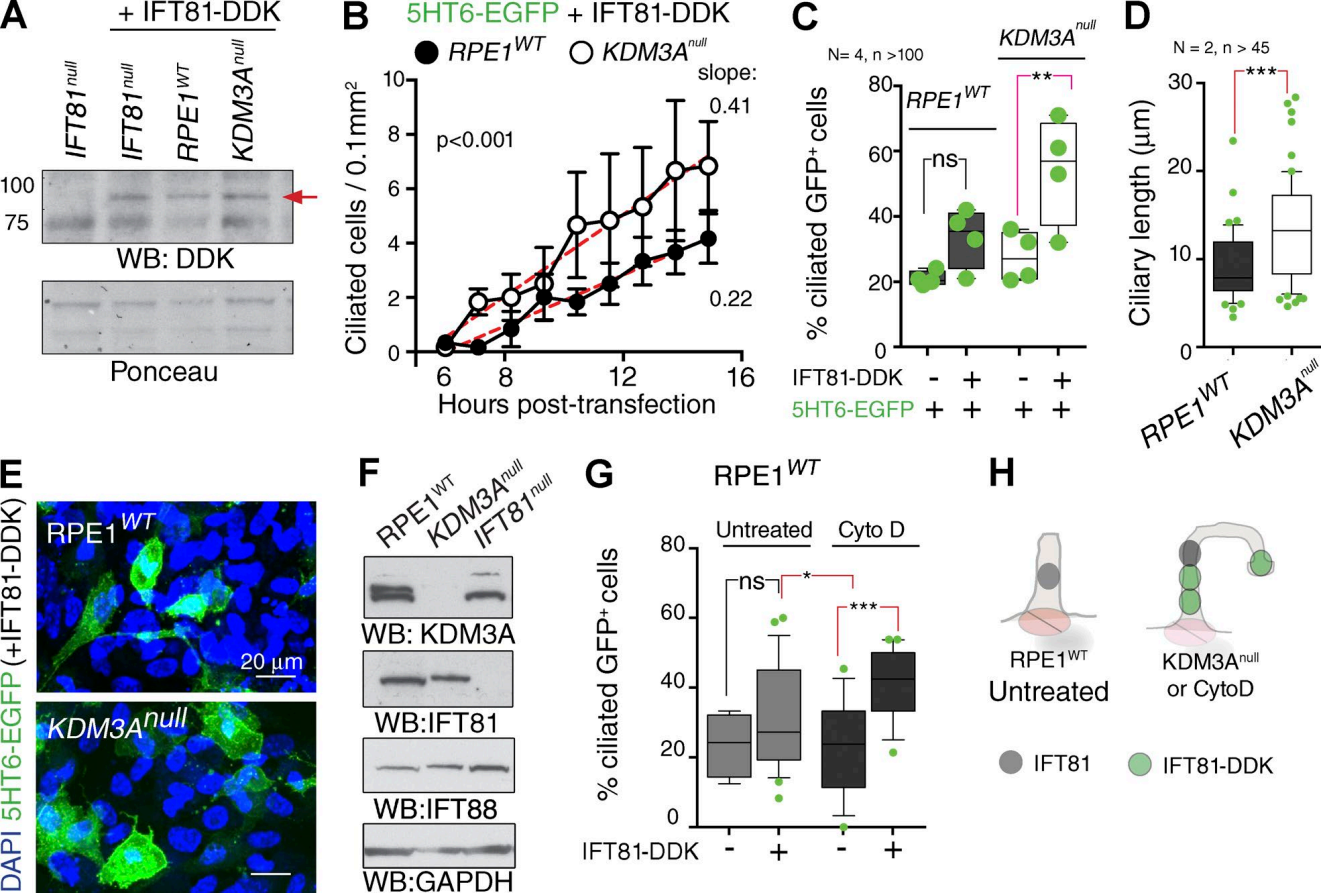
**KDM3A uncovers an actin-mediated control on IFT that precludes IFT81 overexpression.** (A) Immunoblots with DDK antibody of total cell extracts harvested 24 h after transfection with IFT81-DDK plasmid. *IFT81*^*null*^ cells transfected with 5HT6-EGFP used as negative control for DDK signal (red arrow). Ponceau red is a loading control. (B) Line graph showing the number of 5HT6-EGFP–positive cells with a cilium at the indicated time points. Error bars represent SEM between fields of view. Linear regression and p-values from comparing the individual slopes by paired *t* test. See also Fig. S3 (E and F). (C) Median and range of the percentage of 5HT6-EGFP–positive cells that contain a cilium under each experimental condition. Each dot is the percentage of one independent experiment (*N* = 4 transfections, *n* > 100 GFP+ cells scored/condition). **, P < 0.01 (paired *t* test). (D) Mean ± 10th–90th percentile of lengths of 5HT6-EGFP–positive ciliafrom the two last cotransfections with IFT81-DDK presented in C. ***, P < 0.001 (paired *t test*). (E) Plates fixed after live imaging confirm equal cell densities in *RPE1^WT^* and *KDM3A^null^* cultures. (F) Immunoblots of total cell extracts from the indicated wild-type and mutant lines after 3 h −FCS (ciliary growth) indicate comparable total levels of endogenous IFT components. (G) Mean ± 10th–90th percentile of *RPE1^WT^* cells cotransfected with 5HT6-EGFP and vector control (−) or IFT81-DDK plasmid that contain a cilium measured 24 h after transfection in the presence or absence of 0.5 µM CytoD. Each dot is the percentage of one field of view taken (*N* = 2 transfections, *n* = 4, 28, 12, and 20 fields scored/condition). Analysis of variance: P < 0.004; *t* test for the indicated paired comparisons: *, P < 0.05; ***, P < 0.0005. (H) Graphical summary illustrates the insensitivity of *RPE1^WT^* to IFT81 overexpression unless actin is depolymerized or KDM3A is absent. ns, not significant.

We generated a functional IFT81-DDK construct capable of rescuing ciliogenesis in an *IFT81*-null RPE1 line (Fig. S3, A–D). Comparable transfection levels of IFT81-DDK onto *RPE1^WT^* and *KDM3A^null^* cells were confirmed by immunoblotting (Fig. 7 A). Live imaging revealed that overexpressing IFT81 enabled formation of more cilia and with faster kinetics in *KDM3A^null^* mutants compared with *RPE1^WT^* cells (Fig. 7 B, slopes; and Fig. S3, E and F). Importantly, expression of 5HT6-GFP alone did not recapitulate this increased rate of ciliogenesis in *KDM3A^null^* cells (Fig. 7 C), demonstrating that this difference is not simply caused by an increased propensity to ciliate but is dependent on ectopic IFT81. In the presence of IFT81-DDK, *KDM3A^null^* cilia are also longer than controls (Fig. 7 D). These results did not reflect differences in cell density (Fig. 7 E) or levels of endogenous IFT-B components (Fig. 7 F). This indicates that increasing the abundance of IFT promotes ciliogenesis in *KDM3A* mutants, but not wild-type controls.

Because CytoD treatment of control cells phenocopied the ciliary traits of *KDM3A^null^* mutants, we simultaneously induced actin depolymerization along with IFT81 overexpression in *RPE1^WT^* cultures. Indeed, this dual treatment allows wild-type cells to become sensitive to IFT-B overexpression when actin is depolymerized (Fig. 7 G).

**Figure 8.**
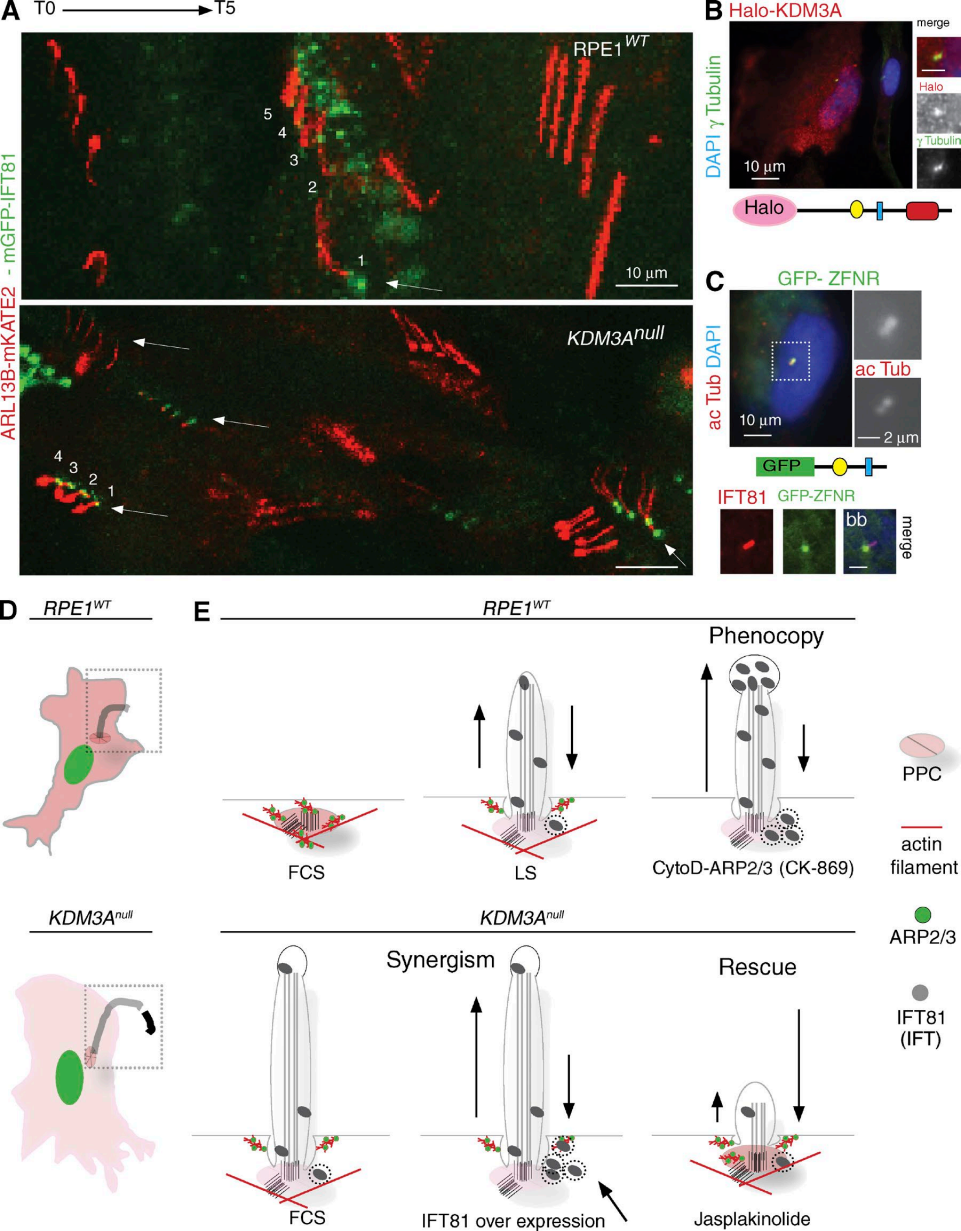
**Proposed mechanism for KDM3A in ciliary regulation.** (A) Collapsed time frames (3 h) of live *RPE1^WT^* and *KDM3A^null^* cultures transiently transfected with ARL13B-mKate2 and IFT81-mGFP showing consistently (arrows) increased accumulations and/recruitment of IFT81-EGFP at the mutant basal body. (B) Enrichment of Halo-KDM3A in centrosomes in ∼2% of transfected cells. (C) Centrosomal localization of KDM3A confirmed by construct containing its zinc-finger and nuclear receptor–binding domains fused to GFP, colocalizing with IFT81. See also Video 5. These findings support KDM3A regulating both global and local actin dynamics at specific subcellular compartments with a direct impact on ciliogenesis. (D) Summary of *KDM3A* mutant cellular phenotype: In the absence of KDM3A, the actin cytoskeleton contains reduced abundance of actin filaments, wide lamellipodia at all cellular edges and reduced migration. Without an actin gate, ciliogenesis is facilitated, and these cilia are long but unstable. (E) Summary of serum (+FCS or low serum [LS]) and pharmacogenetic modulation of actin at the pericentriolar periciliary compartment (PPC) illustrates that promoting actin polymerization either phenocopies ciliary growth in wild-type cells or rescues the abnormal resorption of *KDM3A* mutant cilia. CK-869 treatment of cells pinpoints the actin nucleation activity of ARP2/3 to be necessary to maintain normal IFT within cilia. IFT81 accumulations suggest unbalanced anterograde-retrograde transport (represented by different arrow size) when actin is perturbed. Together, these results indicate that the ciliary traits of *KDM3A* mutants stem from perturbations of actin dynamics, which upset IFT.

Together, these findings provide further and independent evidence of deregulated IFT in *KDM3A* mutant cilia that result from perturbations in actin dynamics. When actin is unperturbed, increasing IFT pools in control cells makes no difference to ciliary growth, indicating that actin allows wild-type cells to maintain tight regulation of IFT entry into the ciliary compartment (Fig. 8 A). In contrast, when actin nucleation is perturbed either genetically (*KDM3A* mutants) or chemically (CytoD treatment ARP2/3 inhibition), this constraint is lifted.

## Discussion

Our results show that KDM3A plays a role in mammalian ciliogenesis through the modulation of actin dynamics. In the absence of KDM3A, the assembly and growth of primary cilia is enhanced. However, cilial stability is compromised such that the length of mutant axonemes and presence of IFT proteins within cilia become unregulated. We find that *KDM3A^null^* cells have reduced abundance of actin filaments. This is because KDM3A plays a transcriptional role regulating actin gene expression and binds directly to actin protein, influencing local actin networks. We demonstrate that the ciliary traits uncovered in *KDM3A* mutants can be chemically reversed or phenocopied in wild-type cultures by altering actin dynamics. Through independent approaches involving genetics, small-molecule inhibitors, and IFT overexpression, we uncover an actin-mediated constraint on IFT within mammalian cilia. Our findings uncover a functional interaction between the actin cytoskeleton and IFT that is perturbed in the absence of KDM3A.

Cells adapt to new environments, like changes in the serum content of the media, through simultaneous changes in gene expression, cytoskeletal rearrangements, and cilia signaling. This integrated response requires signal transduction relays that communicate the cytoplasmic actin polymerization status with IFT in cilia and histone code in the nucleus. Through the use of *KDM3A* mutants, we have uncovered a requirement for KDM3A, which acts as a nexus in this intracellular signaling network. RNA sequencing and proteomic profiling reveal a transcriptional role for KDM3A in increasing the pool of actin in response to serum replenishment that is concomitant with actin filament formation and ciliary resorption. Failure of *KDM3A* mutants to up-regulate actin expression will thus likely contribute to the poor resorption of mutant cilia. During disassembly, formation of new actin filaments at the ciliary base would likely require a pool of free actin to restrict access into the ciliary compartment. Nevertheless, in the absence of serum *KDM3A^null^* mutants contain comparable levels of actin transcripts and protein to those found in control cells, yet it is under these growth conditions that ciliary extension is facilitated in *KDM3A* mutants. Here, we propose that direct protein interaction of KDM3A with actin locally creates an actin gate at the ciliary base (Fig. 8, B and C; and Video 5) to regulate entry of IFT. In the absence of KDM3A, access to cilia is unrestricted and balance and/or kinetics of IFT are perturbed.

The fact that KDM3A is an enzyme that binds actin through its catalytic domain, together with previous observations that actin proteins could be abnormally methylated in *Kdm3a* mutant testes (Kasioulis et al., 2014), prompted us to test the demethylase activity of KDM3A in lysine-methylated actin peptides found in this study. However, as yet we have failed to detect changes in methylation by *KDM3A* in any tested peptide in vitro (unpublished data). Although it remains to be seen whether KDM3A can posttranslationally modify actin in vivo, the binding of KDM3A to actin itself is likely to modulate binding of other actin modifiers, including ARP2/3 and the capping components, to directly tune actin networks.

The functional interplay between KDM3A-ARP2/3 is interesting; *KDM3A^null^* cells respond to lower CK-869 doses than *RPE^WT^* cells, suggesting that the ciliary traits of *KDM3A* mutants have already compromised ARP2/3 function. A role for ARP2/3 at the cilium/centrosome has been suggested. ARP2/3 mediates actin nucleation at centrosomes (Farina et al., 2016). Silencing of *ACTR3* or *ARP2* in cultured cells promotes ciliogenesis but with unclear kinetics, as cells were phenotyped 2.5–3 d after siRNA depletion was initiated (Kim et al., 2010; Cao et al., 2012). In our study, we used a small-molecule inhibitor, CK-869, which prevents the formation of new actin filaments and unlike CytoD does not destabilize filaments that are already formed (Hetrick et al., 2013). We demonstrate that acutely blocking ARP2/3 activity (3-h treatment) alters the distribution of endogenous IFT-B within cilia. This suggests that continual actin nucleation by ARP2/3 is required for regulated entry of IFT into cilia and cilial length maintenance.

Our identified roles for KDM3A in actin dynamics have further implications. Understanding how cancer cells can subvert actin bundling during metastasis is an emerging and exciting theme in research. Although the brunt of the current focus of overexpression of KDM3A in various types of cancer has been on its transcriptional targets (Krieg et al., 2010; Uemura et al., 2010; Cho et al., 2012; Osawa et al., 2013; Tee et al., 2014; Parrish et al., 2015), our work suggests that cytoplasmic KDM3A may act as an important cytoskeletal rheostat integrating mechanosensation (Kaukonen et al., 2016; Pedanou et al., 2016) with cell adhesion and signaling through cilia. Aberrant length ranges and resorption of *KDM3A* mutant cilia in response to environmental cues could also contribute to metabolic imbalance in *Kdm3a* mouse models (Okada et al., 2010), as ciliary length is tightly regulated in response to leptin levels (Han et al., 2014).

Our findings that KDM3A has roles beyond the “direct” regulation of transcription (i.e., acting as an H3K9 demethylase; Yamane et al., 2006) links cytoplasmic sensing of actin polymerization, ciliogenesis, and transcriptional activity in response to serum. KDM3A is a versatile protein that is tightly regulated in response to environmental inputs (Pollard et al., 2008; Krieg et al., 2010; Cheng et al., 2014; Kasioulis et al., 2014; Abe et al., 2015) with both catalytic and structural roles (Abe et al., 2015; Schneider et al., 2015) involving binding to histone (Yamane et al., 2006) and nonhistone proteins (Ramadoss et al., 2017) within nuclear and cytoplasmic compartments (Okada et al., 2007; Yang et al., 2009; Yamada et al., 2012; Kasioulis et al., 2014; Kaukonen et al., 2016). KDM3A thus appears to be capable of integrating epigenetic and cytoskeletal regulation with ciliary IFT in response to extracellular cues. 

While our manuscript was in press, actin-mediated scission of the tips of mammalian primary cilia was uncovered as a mechanism to clear activated ciliary receptors (Nager et al., 2017) or to promote ciliary disassembly (Phua et al., 2017), independent of retrograde IFT involvement. The behavior of *KDM3A^null^* cilia is consistent with these findings; through its role modulating actin dynamics, KDM3A would also modulate ciliary ectocytosis at the tip. The relative contribution of unrestricted IFT recruitment to cilia or reduced IFT clearance through ectosytosis in KDM3A mutants is an exciting topic for future research.

## Materials and methods

### Plasmids

Plasmids encoding the serotonin receptor pEGFPN3-5HT6 (Berbari et al., 2008), IFT81-DDK, and Halo-KDM3A were purchased from Addgene (35624), OriGene (RC216437), and Kazusa DNA Res Institute (FHC05559), respectively. IFT81-mGFP was made by transferring Sgf1-Mlu1 restriction fragment from clone RC216437 into mTagGFP vector PS100048 (OriGene). The KDM3A deletion mutant GFP-ZFNR was cloned using PCR primers containing Bgl2 and Kpn1 sites for cloning into EGFP-C1 vector (Takara Bio Inc.; listed in Table S1) with Halo-KDM3A as template and PfuUltra II DNA polymerase (Agilent Technologies), subsequently confirmed by sequencing. KDM3A_i2_-EGFP (Kasioulis et al., 2014) contains all the functional domains of full-length KDM3A except for a nuclear localization signal (Brauchle et al., 2013).

### *Kdm3a* mouse model

The deletion mutant (*Kdm3a^ΔJC^*) and hypomorphic gene-tarp (*Kdm3a^GT^*) mouse models were described previously (Tateishi et al., 2009; Kasioulis et al., 2014). Genotyping was done with PCR primers as in Table S1.

### Cell lines and CRISPR/Cas9 mutants

Primary MEF cultures were established by mincing embryonic day 11.5–13.5 embryos in DMEM supplemented with antibiotics, FCS, and β-mercaptoethanol and grown in 3% O_2_. Telomerase immortalized human retinal pigment epithelial cells (hTERT-RPE1, referred to here as *RPE1^WT^*) were from Takara Bio Inc. and maintained in DMEM/F-12 (1:1; Thermo Fisher Scientific). *RPE1^WT^* cells were engineered with a double nicking strategy using CRISPR/Cas9 nickases following reported guidelines (Ran et al., 2013). Oligonucleotides to produce guide RNAs (listed in Table S1) were designed with CRISPR Design Tool (http://tools.genome-engineering.org). In brief, guides cloned onto pX461 or pX462 (Addgene) mammalian expression plasmids were cotransfected onto *RPE1^WT^* cells followed by 24 h of 3 µg/ml puromycin selection and individual GFP-positive cells sorted by FACS onto 96-well plates to establish the RPE1 lines *KDM3A^null^* and *IFT81^null^*. *RPE1^CrWT^* derives from noneventful CRISPR targeting of *IFT81,* subsequent sequencing confirmed wild-type alleles for both IFT81 and KDM3A. Mutations were confirmed by immunoblotting with KDM3A and IFT81 antibodies and sequencing of genomic PCR (primers are listed in Table S1) cloned into pGEM-T Easy (Promega) using T7 and SP6 primers.

### Transfections

Parental and engineered RPE1 lines were transfected with Lipofectamine2000 (Thermo Fisher Scientific) following the manufacturer’s instructions, grown without antibiotic the day before, and transfected in the absence of serum for 5 h. All cotransfections were done mixing the DNA of the relevant cotransfected plasmids before the addition of transfection reagents.

### Antibodies

Antibodies were from the following sources: *N-*KDM3A (12835; Proteintech), *C-*KDM3A (NB100-77282; Novus Biologicals), anti–acetylated α-tubulin (T7451; Sigma-Aldrich), γ-tubulin (GTU-88; Sigma-Aldrich), IFT88 (13967-1-AP; Proteintech), IFT81 (11744-1-AP; Proteintech), GAPDH (5019A-2; ImGENEX), ARL13B (17711-1-AP; Proteintech), DDK tag (TA50011; OriGene), and Halo (G9281; Promega). Secondary antibodies for immunofluorescence and immunoblotting are Fab′2 IgG Alexa Fluor from Molecular Probes and HRP conjugated from EMD Millipore and Sigma-Aldrich.

### Drug treatments

CytoD (25023; EMD Millipore), CK-869 (C9124; Sigma-Aldrich), and jasplakinolide (1689-05; BioVision) were dissolved as recommended by the manufacturers and used at the indicated concentrations. Controls for each drug treatment contained the relevant solvent only.

### Ciliary phases

For the study of cilia dynamics, cells were plated 24 h in advance. In all cases, stocks of all cell lines were maintained at comparable cell densities. For the studies of ciliogenesis in exponentially growing cultures, cells were not allowed to reach confluency during two consecutive passages before assaying ensuring a consistent number of cycling cells. For cilia resorption studies, cells were plated between 40% and 50% confluency followed by a further 24, 48, or 72 h culture without serum (−FCS, as indicated in each panel); for ciliary growth assays (3–24 h), cells were plated at 60–70% confluency. Resorption was induced by replacing serum-free media with media containing 10% FCS (+FCS) for the indicated times. Temperature-induced resorption was done by adding 10% FCS and floating the tissue culture plates on a water bath maintained at 42°C for the indicated times.

### Cell spreading assays

Actively growing cultures were trypsinized and resuspended in complete media, replated immediately, or spun down and placed in suspension without serum for 90 min in a cell incubator. Replated cells were imaged 90–120 min after replating on uncoated plastic plates.

### Yeast two-hybrid studies

The initial yeast two-hybrid screen was performed using an embryonic eye mouse cDNA library (Kasioulis et al., 2014) with the JMJC domain of KDM3A as bait cloned through partial EcoRI digestion into the DNA-binding domain encoding plasmid pGBKT7. Bait and prey plasmids from blue colonies growing in quadruple selection (Leu/Trp/Ade/His) were rescued by cell lysis and bacterial transformation in ampicillin (pGADT7) or kanamycin (pGBKT7) plates and sequenced. Plasmids encoding CAPZB and ACTA2 were cotransformed back into the yeast two-hybrid Gold strain (Takara Bio Inc.) along with JMJC-pGKBT7, as described in the Matchmaker two-hybrid system protocol. Independent colonies grown in double selection (Leu/Trp) where resuspended, and a 1/100 dilution was replated in 0.2 µg/ml aureobasidin A double selection plates in the presence of X-α-Gal (Takara Bio Inc.).

### Immunofluorescence

Cells were grown on glass coverslips and fixed (10 min, 4% methanol-free formaldehyde), except for those shown in Fig. 6 (C and D), which were fixed with ice-cold methanol for 5 min. Fixed cells were permeabilized (15 min in 0.25% Triton X-100) and blocked in 2% BSA before incubation with antibodies and DAPI and mounted in Prolong Gold (Thermo Fisher Scientific). Rhodamine–phalloidin (R415; Thermo Fisher Scientific) added to the secondary antibody incubation was used to visualize F-actin of fixed cells.

### Imaging

Live-cell microscopy was done using glass-bottom plates (662892; Greiner Bio-one) in a heated chamber with a source of nitrogen using a 40× plan Fluor 0.75 NA dry lens in an A1R confocal microscope (Nikon) in the relevant culture media for each growth condition. Movies shown were acquired as to minimize photobleaching. Postacquisition analyses were done with NIS-Elements (Nikon) on maximum-intensity projections using Fiji (ImageJ) and IMARIS 8.4 (Bitplane). Ciliary length studies on fixed cells were done with wide-field microscopy or confocal imaging as indicated in each panel using a 40× dry lens and FIJI software for measurements.

### Cell lysis and immunopurifications

Total extracts for immunoblots were obtained by lysing cells in 150 mM Tris-HCl, pH 7.5, 50 mM NaCl, 0.5% IGEPAL, 1% Triton X-100, 1 mM EDTA, 0.5% deoxycholate, Benzoase DNase (EMD Millipore), and Halt protease inhibitor (1860932; Thermo Fisher Scientific). Total cell extracts for total cell proteome were done in PBS and 1% SDS in the presence of protease inhibitor, sonicated, and cleared by 10-min centrifugation at 14,000 rpm. For KDM3A interactome, *RPE1^WT^* (in duplicate), *RPE1^WTcr^*, and *KDM3A^null^* (line 2, in triplicate) cells were plated the day before and grown in the absence of serum for 3 h, lysed in 50 mM Tris-HCl, pH 7.5, 100 mM NaCl, 10% glycerol, 5 mM EDTA, 0.5% IGEPAL, and protease inhibitors and incubated with N-KDM3A antibody (12835; Proteintech) for 2 h at 4°C followed by 15-min incubation with PureProteome magnetic beads (EMD Millipore) to concentrate the immunoglobulin complexes. Three successive washes were performed to reduce the IGEPAL concentration.

### Far–Western blot

Serial dilutions (50–200 ng/µl) of pure actin derived from rabbit skeletal muscle (AKL99; Cytoskeleton, Inc.) resolved in 4–12% acrylamide were transferred onto nitrocellulose and stained with Ponceau red to visualize loadings. Bacterially expressed GST or GST-JMJC immobilized in glutathione magnetic beads (8821; Proteintech) were washed and eluted in glutathione buffer. Membrane blocked with 5% BSA was incubated with eluted GST proteins in 10 mM Tris, pH 7.6, 100 mM KCl, 1 mM MgCl_2_, 0.1 mM EDTA, 1 mM DTT, 0.1% (vol/vol) Tween-20, and 2% BSA for 1 h. Subsequent steps followed standard immunoblotting protocol using GST antibody to detect GST proteins retained on membrane.

### Mass spectrometry

On-bead purified complexes were digested with trypsin, reduced, alkylated, and desalted, and two runs of each sample were analyzed by liquid chromatography tandem mass spectrometry as described previously (Turriziani et al., 2014). Datasets can be found in *ProteomeXchange* identifier PXD004334. Data were analyzed using the MaxQuant software suite by searching against the UniProt HUMAN database with protein N terminus (acetylated), M (oxidation). Label-free quantification values were determined by MaxQuant as described previously (Cox et al., 2014). In the search for posttranslational modifications, databases were also searched for peptides with mass modifications as follows: mono-, di-, and trimethylation (delta mass of 14.0157, 28.0313, and 42.04695 D, respectively).

### RNA sequencing

RNA was isolated from RPE1*^WT^* and *KDM3A^null^* (line 2) cells in triplicate cultures under ciliary growing or resorption conditions (Fig. 3 A) using RNeasy Plus (QIAGEN) according to the manufacturer’s instructions. RNA integrity score was calculated with RNA 6000 Nano reagent (Agilent Technologies) in a 2100 Bioanalyzer (Agilent Technologies). All samples used for the preparation of the RNA-sequencing library had a RIN (RNA Integrity Value) score above 9. Library construction used poly(A) RNA selection, and all 100-bp reads were obtained using Illumina HiSeq2000. Cufflinks, CummeRbund, and Picard were used for alignments to reference genome, transcript assembly, quantification, and statistical analysis, respectively, done at Oxford Gene Technology (The Molecular Genetics Company). The mean read depth for each sample was of 13 × 10^6^.

### GO term analyses

Genes or proteins differentially enriched in proteome or interactome datasets were analyzed using DAVID (the Database for Annotation Visualization and Integrated Discovery).

### Statistics

Statistics were done using GraphPad Prism 5 software. One-way analysis of variance was used for the initial assessment of multiple comparisons. In those cases where a significant difference was observed (P < 0.01), a pairwise two-tailed Students *t* test was applied to compare individual conditions. χ^2^ *t* test was applied to compare percentages as indicated in each panel. Asterisks indicate p-values (*, P < 0.05; **, P < 0.01; ***, P < 0.001; ****, P < 0.0001) calculated through the statistical analysis indicated in each case. N represents the number of experimental or biological replicates, and n represents the number of events measured in all experimental or biological replicates as indicate.

### Online supplemental material

Table S1 shows sequences of primers used to clone *KDM3A* expression plasmids, CRISPR/Cas9 editing, and all genotyping. Table S2 contains raw values of RNA sequencing reads and proteomic studies. Table S3 shows thresholding criteria and the resulting lists of genes that were subsequently used for GO term analysis. Table S4 shows enriched GO terms. Fig. S1 shows genotypes of *KDM3A^null^* cells, ciliary anomalies of *Kdm3a^GT/GT^* hypomorph MEFs, and effect of overexpressing KDM3A in the percentage of ciliated cells. Fig. S2 shows phalloidin staining of *Kdm3a^ΔJC/ΔJC^* MEFs, assessment of KDM3A interaction with actin by two-hybrid screening and far–Western blotting, and detailed measurement criteria used to quantify the extent of filopodia in transfected cells. Fig. S3 shows genotyping of *IFT81^null^* cells, functionality of IFT81-DDK construct, and identical transfection efficiencies of 5HT6-EGFP+IFT81-DDK onto *RPE1^WT^* and *KDM3A^null^* cells. Videos 1 and 2 show instability of *KDM3A^null^* cilia. Video 3 shows instability of *Kdm3a^ΔJC/ΔJC^* cilia. Video 4 illustrates the unresponsive behavior of *KDM3A^null^* cilia to serum-induced resorption. Video 5 illustrates that KDM3A_i2_-EGFP can be found in cilia.

## Supplementary Material

Supplemental Materials (PDF)

Video 1

Video 2

Video 3

Video 4

Video 5
