# Supplementary material for: KDM3A coordinates actin dynamics with intraflagellar transport to regulate cilia stability
Source: J Cell Biol. 2017 Apr 3;216(4):999–1013. doi: 10.1083/jcb.201607032 (PMC5379941; doi:10.1083/jcb.201607032)
Supplement: Supplemental Materials (PDF) [file JCB_201607032_sm.pdf]

Yeyati et al., https://doi.org/10.1083/jcb.201607032

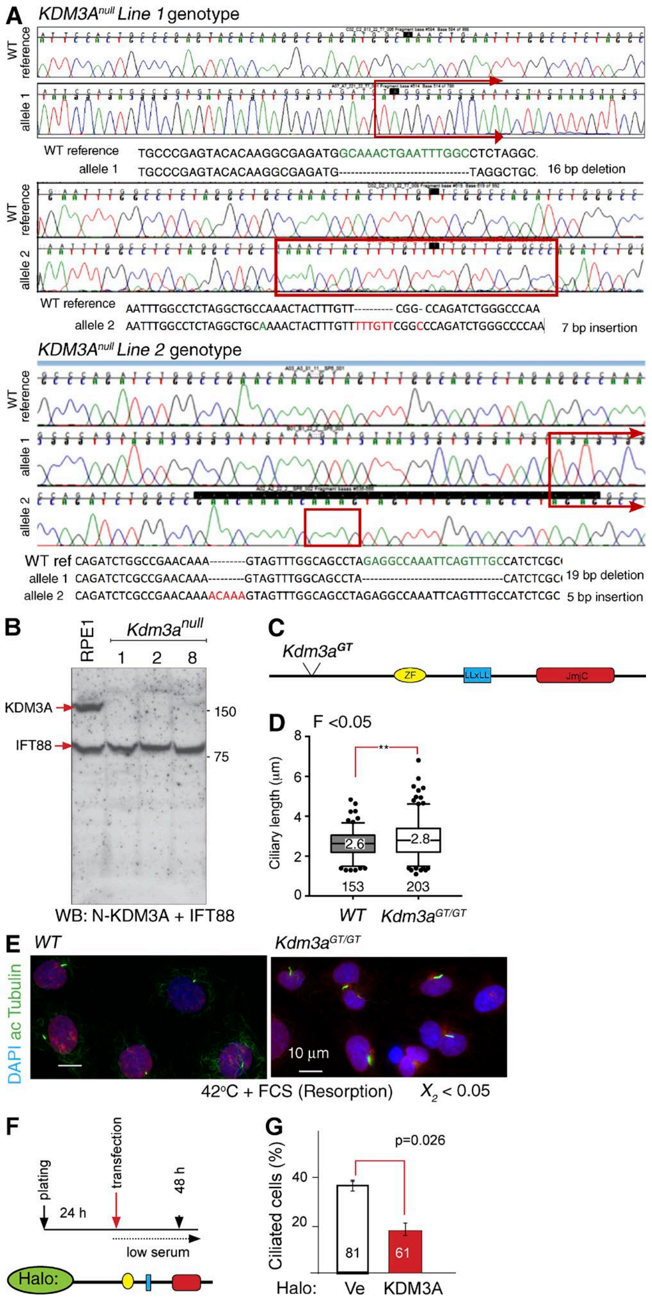

Figure S1. **Genotypes of KDM3A CRISPR lines.** (A) Sequencing indicates that *KDM3A^{null}* lines 1 and 2 are compound heterozygotes with insertions and deletions. WT, wild type. (B) Immunoblots indicate absence of KDM3A (KDM3A red arrow) in *KDM3A^{null}* lines 1, 2 and 8 as shown here and in Fig. 2 A using antibodies directed to the C- and N-terminal end of KDM3A, respectively. An antibody to IFT81 (IFT81 red arrow) was used to here as loading control. (C) Schematic of gene-trap insertion in *Kdm3a^{GT}* mutant mouse model. (D) Mean ± 5th to 95th percentile of MEF ciliary lengths reveal a wider range of lengths in mutants with comparable mean lengths (*N* = 2 embryos/genotype). (E) Immunostaining of MEFs with acetylated-α tubulin show remaining cilia in *Kdm3a^{GT}* after serum and temperature-induced resorption. χ^2 value from >130 cells scored for each genotype. (F) Experimental design and details of KDM3A Halo-tagged construct. (G) Percentage of ciliated cells 48 h after transfection with Halo control vector or Halo-KDM3A. Error bars represent SEM (*N* = 3 replicates). **, P < 0.01, *t* test analysis.

THE JOURNAL OF CELL BIOLOGY

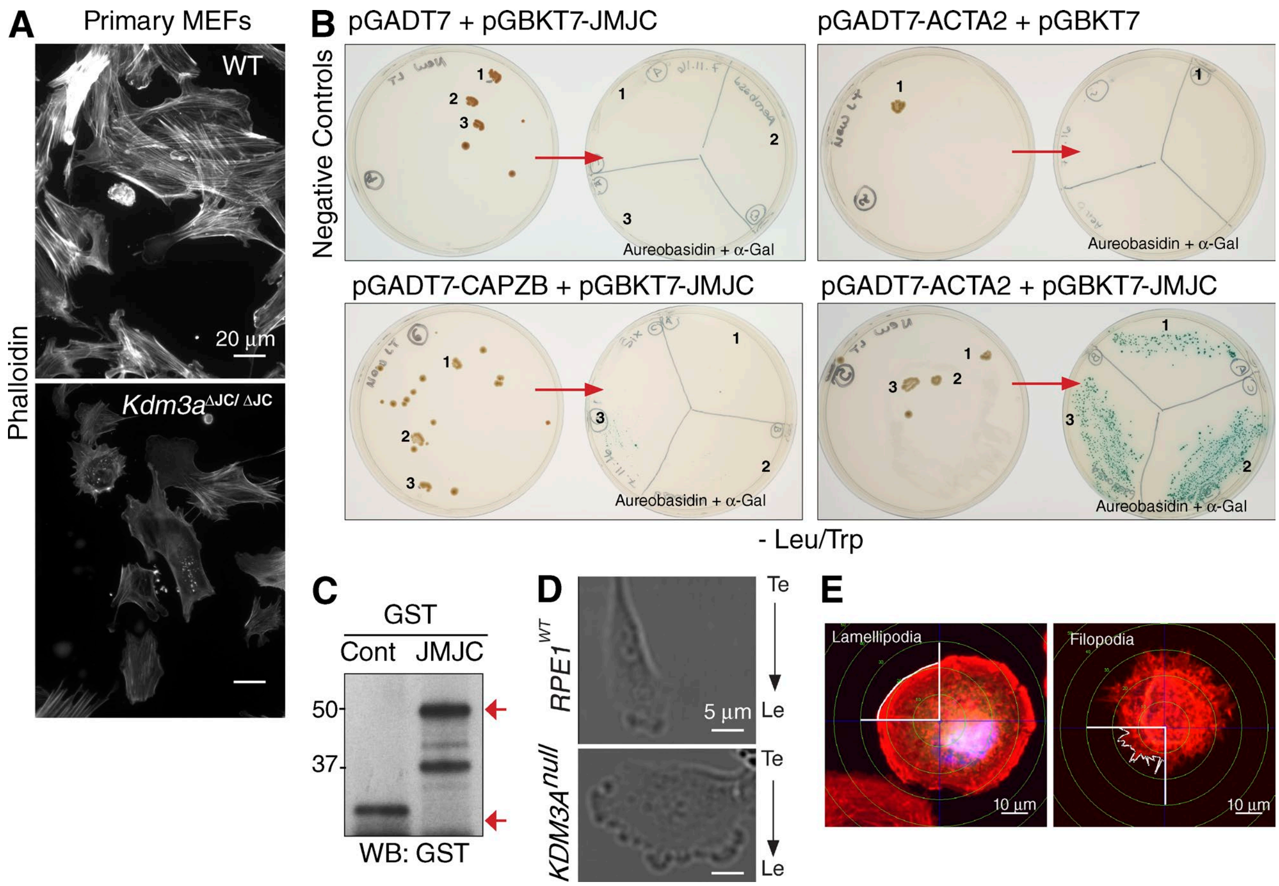

Figure S2. **Effect of KDM3A on actin and actin related phenotypes.** (A) Phalloidin staining of wild-type (WT) and *Kdm3a*$^{\Delta JC/\Delta JC}$ MEFs maintained in low serum for 24 h. (B) Plates showing colonies of bait/prey plasmid cotransformed onto yeast two-hybrid Gold (Takara Bio Inc.) grown in double selection (Leu/Trp). The indicated colonies were resuspended and replated (red arrows) in double selection in the presence of aureobasidin and X-α-Gal. Only yeast cotransformed with JMJC and ACTA2 were able to consistently grow under stringent antibiotic selection indicating a binary interaction of KDM3A with actin, but not CAPZB. (C) GST fusion proteins incubated with nitrocellulose membranes containing actin. (D) Still frames of migrating cells 3.5 h after attachment show wide leading edge (Le) in *KDM3A*^{null} cells and pointed leading edge in wild-type controls. Te, trailing edge. (E) The length of the line drawn following the cellular edge contained in the concentric circle with a radius of 20 µm (measurement strategy used in Fig. 4 J) was used to quantify filopodia-like projections in Halo transfected cells.

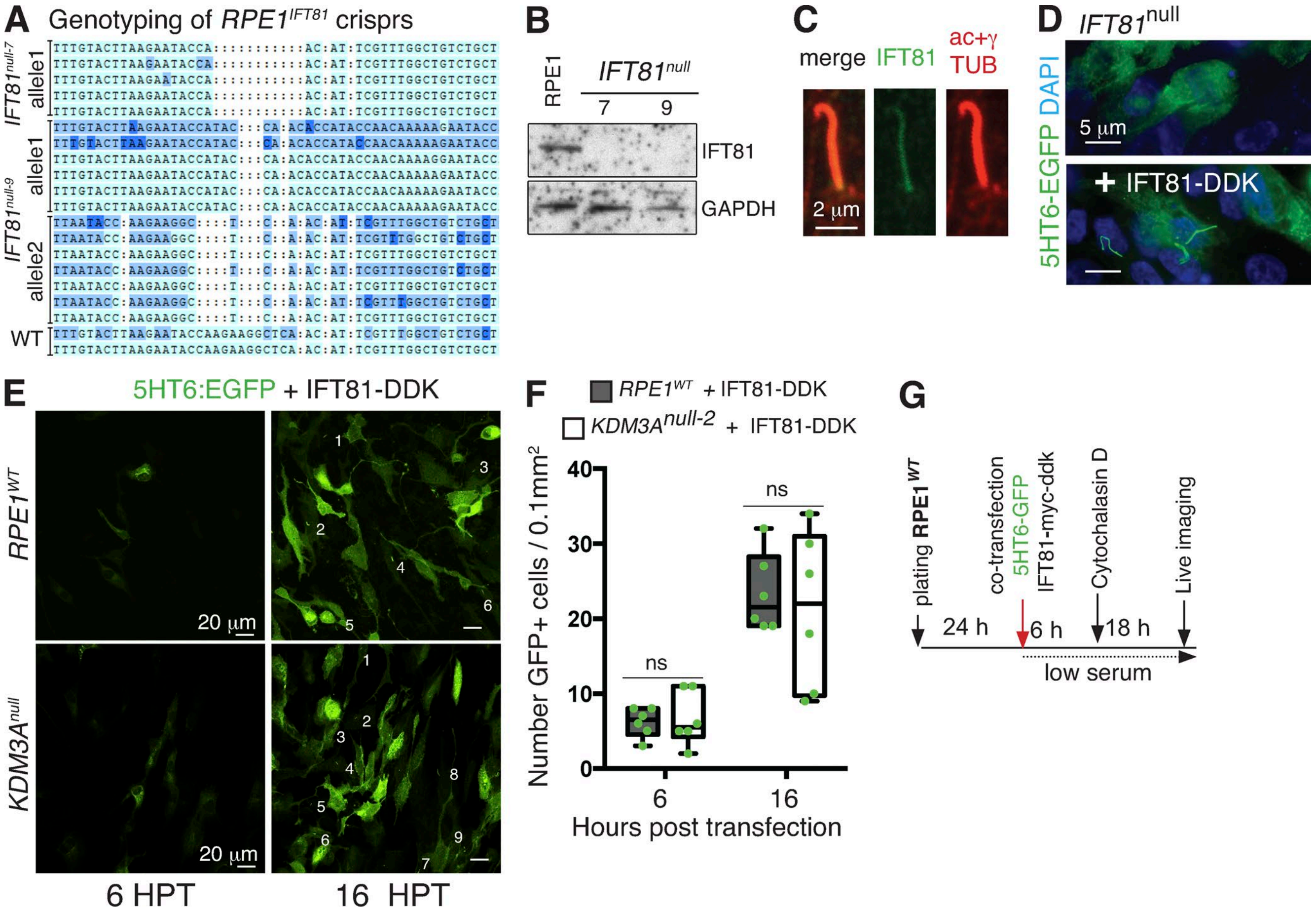

Figure S3. **A correlation between *KDM3A* genotype and IFT.** (A) Genotype of $IFT81^{null}$ mutants generated by CRISPR/Cas9. (B) Immunoblot of total extracts shows absence of IFT81 in $IFT81^{null}$ lines. (C) IFT81-DDK rescues of the absence of cilia of $IFT81^{null}$ cells, using 5HT6-GFP as ciliary marker. (D) Immunostaining with anti-DDK shows presence of tagged-IFT81 in the rescued cilia of the $IFT81^{null}$ cells. (E) Single time frames showing first (6 h after transfection) and last (16 h after transfection) captures taken during ciliary growth (shown in Fig. 7 B) illustrate number of GFP^{+} cells before and after time captures. Numbers within panels indicate presence of cilia (16 h after transfection). (F) Median and range of GFP^{+} cells per field of view (0.1 mm^2) measured in Fig. 7 B shows comparable numbers of transfected cells between the cell lines. Each dot represents one field of view measured. ns, not significant. (G) Experimental design used in Fig. 7 G for the simultaneous overexpression of IFT81 and actin depolymerization.

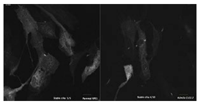

Video 1. **Composite movie showing parental $RPE1^{WT}$ and $KDM3A^{null}$ (line 2, herein named $KDM3A^{cr22.2}$) cells transiently expressing 5HT6-EGFP as ciliary marker.** Capture spans 4 h (approximately one frame every 10 min) from the moment of serum withdrawal (promoting ciliary growth) from confluent cultures. The number of stable cilia relative to the total number of cilia seen in these fields of view is indicated. Bar, 20 µm.

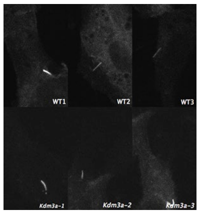

Video 2. **Close-ups from Video 1 showing stable $RPE1^{WT}$ and unstable $KDM3A^{null}$ cilia.** Some still frames are shown in Fig. 6 A.

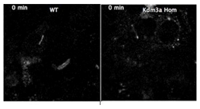

Video 3. **Composite movie showing primary wild-type and *Kdm3a*$^{\Delta JC/\Delta JC}$ MEFs transfected with ARL13B-mKATE2.** Captures show cilia of confluent cultures maintained in the absence of serum.

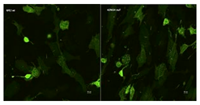

Video 4. **Composite movie shows parental RPE1^{WT} and *KDM3A*^{null} cells transiently expressing 5HT6-EGFP during ciliary resorption (from time frame 5).** Capture spans 115 min (approximately one frame every 3 min), after which most of RPE1^{WT} cilia are resorbed but that of *KDM3A*^{null} remain.

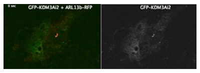

Video 5. **RPE1^{WT} cells cotransfected with KDM3A$_{i2}$-EGFP and ARL13B-mKATE2 (merged channels) show incorporation of tagged KDM3A to the cilium.** The right panel shows the KDM3A$_{i2}$-EGFP single channel.

Table S1. **Sequences of primers used to clone *KDM3A* expression plasmids, CRISPR/Cas9 editing, and all genotyping**

Construct	Forward primer (5′–3′)	Reverse primer (5′–3′)
Expression plasmids		
ZFNR	GAAGATCTTTACCTACAAAGGCTTCTTCTAAGG	CTGGTACCCTAGGTCTACTTCCTGCTCACC
Kdm3a^{null} CRISPR/Cas9 mutants (hTERT- RPE1 human cells)		
Guide 1	CACCGGCCATCTCGCCTTGTGTACT	AAACAGTACACAAGGCGAGATGGCC
Guide 2	CACCGGTACTTTGTTCGGCCAGATCT	AAACAGATCTGGCCGAACAAAGTACC
Ift81^{null} CRISPR/Cas9 mutants (hTERT- RPE1 human cells)		
Guide 1	CACCGGAAGAAGGCTCAACATTCGTT	AAACAACGAATGTTGAGCCTTCTTCC
Guide 2	CACCGGATTCTTAAGTACAAACCTTC	AAACGAAGGTTTGTACTTAAGAATCC
Genotyping of CRISPR/Cas9 mutants		
Kdm3a^{Cr22}	ATGGTAGGCCCTGCTAGAGA	ACTTCTACTCAACAACACAGAACAC
Ift81^{Cr3}	TGATGGGTAGCAGAAAGCCA	GAAGAGAGACAGCATTTTCTCAAA
Genotyping of *Kdm3a*$^{\Delta JC}$ mice (Tateishi et al., 2009)		
a	CCAAATGCCCAAGGCTTTAC	
b		CTGCACCGAATTCTCTTCCG
c		CCACATACACAAACTTCTCCTTAG
Genotyping of *Kdm3a*^{GT} mice (Kasioulis et al., 2014)		
GT1	CTCTGCCCTGTTGGGTCGTGC	
GT2		GATTATCAGTAAGGGAAAGCCGAAGACTG
GT3		CAAGGCGATTAAGTTGGGTAACGCC

Provided online are three Excel tables.

Table S2. Raw dataset values before thresholding. Page 1, table legend; page 2, label-free quantification (LFQ) raw values of total proteome dataset; pages 3 and 4, list of all reads obtained in RNA sequencing from growth (−FCS) and resorption (24 h −FCS, 90 min +FCS); page 5, all label-free quantification values from KDM3A interactome and the imputation applied for subsequent statistical analyses. Full proteomic datasets can also be found in *ProteomeXchange* identifier PXD004334.

Table S3. Thresholded values. Page 1, table legend; pages 2 and 4, RNA-sequencing reads down-regulated ($\log_2$ fold change ≤ −2) in *KDM3A*^{null} cells during ciliary growth (page 2) or resorption (page 4), with a p-value adjusted to account for false discovery rate (Q) ≤ 0.05; pages 3 and 5, list of proteins for which label-free quantification (LFQ) values of *RPE1*^{WT} during ciliary growth (page 3) or resorption (page 5) are two times over that of *KDM3A*^{null} cells with a p-value determined by *t* test ≤ 0.05; page 6, list of proteins immunopurified with an antibody to KDM3A that are enriched fourfold or more (log2 ≥ 2) in *RPE1*^{WT} over *KDM3A*^{null} with a p-value determined by *t* test ≤ 0.05 (represented as $-\log_{10}$ of p for graphical representation in Fig. 3 F).

Table S4. GO term enrichments. Page 1, table legend; pages 2–7, list of all annotated clusters associated with transcripts (transcriptome) and proteins (total proteome) down-regulated in *KDM3A*^{null} cells or significantly enriched in KDM3A pull-downs (interactome).

References

Kasioulis, I., H.M. Syred, P. Tate, A. Finch, J. Shaw, A. Seawright, M. Fuszard, C.H. Botting, S. Shirran, I.R. Adams, et al. 2014. Kdm3a lysine demethylase is an Hsp90 client required for cytoskeletal rearrangements during spermatogenesis. *Mol. Biol. Cell.* 25:1216–1233. http://dx.doi.org/10.1091/mbc.E13-08-0471

Tateishi, K., Y. Okada, E.M. Kallin, and Y. Zhang. 2009. Role of Jhdm2a in regulating metabolic gene expression and obesity resistance. *Nature.* 458:757–761. http://dx.doi.org/10.1038/nature07777
